# A monolithic patient-specific 3D–0D model for In silico investigation of hemodynamics in patients with left ventricular assist devices

**DOI:** 10.1007/s10237-026-02063-9

**Published:** 2026-06-03

**Authors:** Mia Bonini, Marc Hirschvogel, Michael Ferguson, Francis Pagani, Paul C. Tang, David Nordsletten

**Affiliations:** 1 Department of Biomedical Engineering, University of Michigan, Ann Arbor, MI USA; 2 Division 2.2 Process Simulation, Bundesanstalt für Materialforschung und -prüfung (BAM), Berlin, Germany; 3 Department of Cardiac Surgery, University of Michigan, Ann Arbor, MI USA; 4 Department of Cardiac Surgery, Mayo Clinic, Rochester, MN USA

**Keywords:** Patient-specific modeling, Left ventricle assist device, Hemodynamic modeling, Computational fluid dynamics, Lumped parameter modeling

## Abstract

We present a fully coupled, patient-specific 3D–0D computational framework for hearts supported with left ventricular assist devices (LVAD) that enables controlled *in silico* experimentation. The approach monolithically integrates three-dimensional CFD of the left ventricle (LV), left atrium (LA), aortic root, and LVAD cannulae with a closed-loop 0D lumped parameter network of the full circulation. Mitral and aortic valve dynamics are governed by transvalvular pressure and flow with patient-specific regurgitant orifice areas, and the LVAD is represented via a pressure–flow (H–Q) relation. This manuscript provides the complete mathematical formulation, coupling strategy, and parameterization required to build a reproducible pipeline from dynamic CT, 2D transthoracic echocardiography, and right heart catheterization. This methodology is demonstrated in a patient under long-term support of LVAD and concomitant mitral and aortic regurgitation. The personalized, fully coupled 3D–0D models reproduced available clinical targets with a mean error of 8.6%, enabling controlled *in silico* interrogation of valve repair strategies. In the patient-specific state, simulated mitral and aortic regurgitant volumes were 6.6 and 6.5 mL per cycle, yielding a forward cardiac output of 3.16 L/min despite an LVAD flow of 3.7 L/min. In silico isolated mitral valve (MV) repair, isolated aortic valve (AV) repair, and combined MV+AV repair increased forward output to 3.41, 3.33, and 3.55 L/min, respectively; however, aortic valve opening and increased aortic pressure pulsatility (up to 38.9 vs. 13.5 mmHg) were observed only when MV repair was involved. These left-sided improvements propagated through the cardiopulmonary circulation, reducing pulmonary pressures and right ventricular loading, with the largest benefit observed following combined repair. We show that the modeling platform presented provides a powerful means to study mechanical circulatory support, enabling patient-specific evaluation of surgical interventions in patients with LVAD and delivering quantitative insight into clinically important metrics—such as aortic pulsatility, RV afterload, and chamber-level flow patterns.

## Introduction

Heart disease remains the leading cause of death worldwide (Al 1980). In the USA, heart failure accounted for 2.1 million deaths between 2021 and 2024, reflecting a nearly 10% increase in mortality compared to the previous decade (Woodruff et al. 2024). One established treatment for advanced heart failure is the Left Ventricular Assist Device (LVAD), a mechanical pump that supports circulation either as bridge-to-transplant therapy or as destination therapy (Dhamoon 2023). By replacing pulsatile cardiac output with pump-driven continuous flow, LVADs induce profound changes in cardiovascular physiology, including altered valve function, chamber contraction, and chamber filling. While LVADs improve survival and quality of life, serious complications persist. Thrombus formation, right heart failure (RHF), and progressive aortic regurgitation (AR) or mitral regurgitation (MR) have been widely reported in long-term LVAD support (Fatullayev et al. 2015; Bravo et al. 2022; Cowger et al. 2010; Kohno et al. 2025). Clinical metrics for predicting thrombus or RHF remain limited, and studies evaluating comorbid valve regurgitation often reach contradictory conclusions, constrained by small cohorts, selection bias inherent to single-hospital studies, or limited clinical metrics in the dataset (Kohno et al. 2025; Kherallah et al. 2024; Kanwar et al. 2020; Arjomandi Rad et al. 2023; Noly et al. 2022). Moreover, the hemodynamic consequences of progressive AR and MR are not fully characterized, and the benefit of mitral valve repair remains uncertain (Kohno et al. 2025). Current guidelines do not recommend mitral valve repair, despite conflicting evidence, and provide no clear recommendations regarding the optimal timing of intervention for patients with severe aortic regurgitation (Noly et al. 2020). As a result, there is a need for approaches that can systematically evaluate how interventions, such as valve repair, may influence patient-specific hemodynamics and clinical outcomes in hearts with LVAD support.

High-fidelity computational modeling provides a means to investigate these mechanisms, offering access to hemodynamic parameters that are otherwise inaccessible in the clinical setting. Reduced-order approaches based on lumped parameter networks (Chivukula et al. 2024, 2022; Gu et al. 2018; Kar et al. 2005; Gohean et al. 2013) have been particularly effective for exploring system-level interactions, offering efficient simulations of pump speed variation, global pressure dynamics, and ventricular loading conditions. While such models are well suited for capturing bulk cardiovascular behavior, they are less equipped to resolve spatially complex features such as transvalvular flows, localized pressure gradients, or intraventricular residence times. To address these questions, several studies have incorporated three-dimensional fluid and solid mechanics (Grinstein et al. 2021; Kasinpila et al. 2021; Park et al. 2018; Bakir et al. 2018; Ghodrati-Misek et al. 2022; Caruso et al. 2015; McElroy et al. 2020; Sun et al. 2020). These investigations have advanced understanding of LVAD performance in relation to cannula placement, transvalvular flow, arrhythmia, and ventricular contraction. At the same time, practical considerations such as boundary condition specification, wall motion representation, and data availability often require simplifying assumptions, which can limit opportunities for comprehensive validation.

Building on this foundation, our approach integrates a wide range of clinical measurements—including dynamic cardiac CT imaging, right heart catheterization (RHC), and echocardiography—to construct a high-fidelity, patient-specific model that maintains physiological consistency while resolving detailed three-dimensional flow patterns. Here, we present a computational fluid dynamics (CFD) framework for simulating patient-specific hemodynamics in the setting of LVAD support. The model resolves three-dimensional pressure and velocity fields in the left ventricle (LV), left atrium (LA), aortic root, and LVAD inflow and outflow cannulae. Valve opening and closure are governed dynamically by transvalvular pressure and flow, allowing physiologically consistent valve interactions within the 3D domain. Furthermore, we are able to prescribe varying severities of valve regurgitation, as well as model valve repair. This high-resolution CFD model is fully coupled to a lumped parameter network representing the remainder of the cardiovascular system, including the right heart chambers, coronary circulation, and systemic and pulmonary vascular beds. Parameters within the lumped model are tuned to match the patient’s global hemodynamic profile. This coupled approach provides a uniquely powerful platform for probing the interplay between local flow phenomena and global cardiovascular function, enabling mechanistic insights that are not accessible through imaging or lumped parameter modeling alone. In our previous work (Bonini et al. 2025) - a part of the Functional Imaging and Modeling of the Heart conference (FIMH 2025) - we presented an overview of the patient-specific 3D–0D cardiovascular model for hearts with LVAD, focusing on the formulation for governing valve dynamics (using transvalvular pressure and flow and optimization) and 0D parameterization and sensitivity analysis. The present study builds directly upon that foundation in three ways. First, we provide the complete and fully documented mathematical formulation of the LVAD model, including its integration within a coupled multiscale cardiovascular system, thereby enabling reproducibility and transparent reuse. Second, we present a monolithically integrated 3D–0D framework that simultaneously resolves the left ventricle, left atrium, aorta, cardiac valves, and LVAD, with explicit pressure–flow regulation of the valves handled within the coupled system. While individual components of this formulation—such as 3D–0D coupling or valve laws—have appeared in prior studies, existing frameworks rarely include all major left heart compartments and do not address the dynamic interplay of flow and pressure regulation across chambers, valves, and mechanical support in a single monolithic solve. This integrated formulation improves numerical robustness and enables stable simulation of hearts supported by LVAD. Third, we extend beyond the original FIMH case study by performing new controlled in silico analyses of isolated aortic valve repair, isolated mitral valve repair, and combined AV+MV repair, demonstrating how the framework can be systematically applied to interrogate treatment-specific hemodynamic effects under otherwise identical physiological conditions.

This study introduces a patient-specific CFD modeling pipeline to simulate LVAD hemodynamics and to isolate the effects of mitral and aortic valve repair. The methods to do so are described in Sect. 2. Results for the patient-specific case, in silico mitral valve repair, aortic valve repair, and mitral and aortic valve repair are given in Sect. 3. Finally, discussion and conclusion are given in Sect. 4.

## Materials and methods

To demonstrate the capabilities of our computational modeling workflow, we showcase a patient-specific case involving left ventricular assist device support. Section 2.2 describes the construction of the 3D model, while Sect. 2.3 details the coupled 0D representation of the remaining circulation. The governing equations are introduced in Sect. 2.4, and the discretized weak form is presented in Sect. 2.5.

### Medical imaging and patient data

**Fig. 1:**
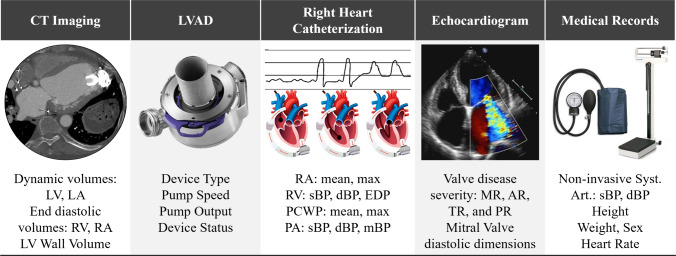
Patient Data collection from showing (left to right) dynamic CT images (giving anatomy, chamber volumes over time), LVAD information (device type, pump speed, total output, whether there is outflow graft obstruction), right heart cardiac catheterization (providing pressures), echocardiogram (valve regurgitation severity), and patient medical records. *Abbreviations:* *LV*, left ventricle; *LA*, left atrium; *RV*, right ventricle; *EDP*, end diastolic pressure; *RA*, right atrium; *RA*, right atrium; *sBP*, systolic blood pressure; *dBP*, diastolic blood pressure; *PCWP*, pulmonary capillary wedge pressure; *PA*, pulmonary artery; *mBP*, mean blood pressure; *MR*, mitral regurgitation; *AR*, aortic regurgitation; *TR*, tricuspid regurgitation; *PR*, pulmonary regurgitation; *Syst.*, systemic; *Art.*, arteries

Clinical data were collected, including dynamic contrast-enhanced computed tomography (CT), noninvasive blood pressure readings, catheter-based hemodynamic measurements, and a 2D transthoracic echocardiographic study (Fig. 1). More information about the data is found in Appendix A. CT scans were acquired using a 64-slice helical scanner (Siemens Somatom Force) with spatial resolutions of 0.4886 mm (sagittal), 0.4885 mm (coronal), and 1.25 mm (axial). Image acquisition occurred during contrast infusion with iopamidol and employed retrospective ECG gating to reconstruct 10 cardiac phases for improved temporal resolution. The patient modeled was a 63-year-old female on a LVAD support for 1.3 years. Device-specific information, including LVAD type (Heartmate 3, Abbott Labs, Chicago, IL), operational speed (5400 rpm), and pump output (3.7 L/min), was incorporated to accurately model left heart and aortic hemodynamics under mechanical support. All data collection and analysis were conducted under IRB-approved protocol HUM00196629 (approved April 2021).

### Anatomic reconstruction and blood flow segmentation

**Fig. 2:**
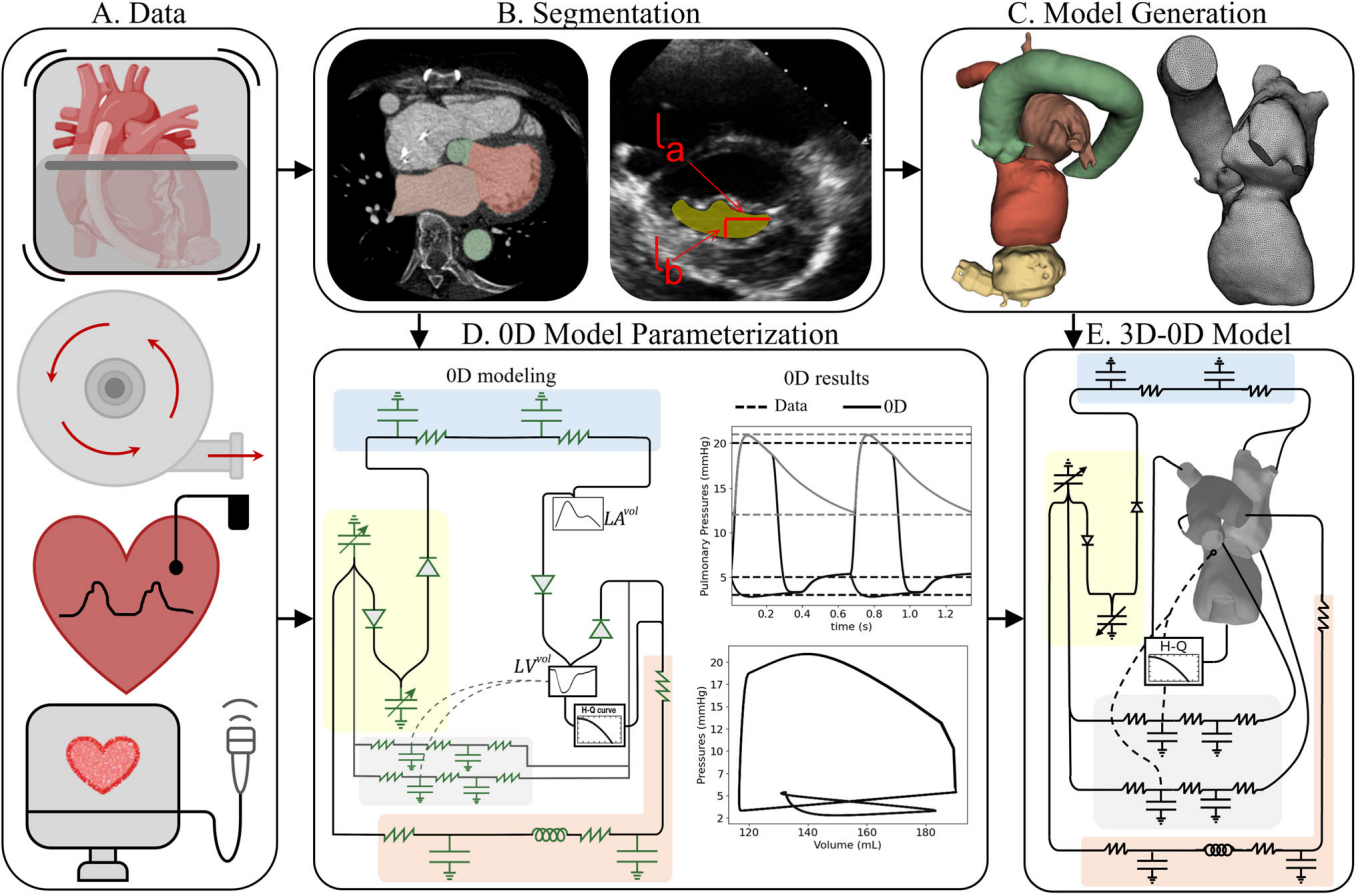
Modeling pipeline **a** data collection (dynamic CT imaging with contrast; LVAD device type, speed, and flow; right heart catheterization; echocardiogram), **b** segmentation (LV, LA, aorta, LVAD cannula) and manual editing in 3D slicer of dynamic CT images and measurement of MV diastolic orifice dimensions from echocardiogram images. **c** Tetrahedral volume meshing was performed in Simmodeler. **d** 0D parameter optimization using patient data. Here we show a schematic of the 0D model on the left and example results on the right where the solid lines are the time-varying 0D results, and the dashed lines are the patient data trying to be matched. **e** 3D–0D CFD modeling. For the 0D model and the 3D–0D model, the systemic system is shaded in orange, the right heart is shaded in yellow, the pulmonary circulation is shaded in light blue, and the coronary system is shaded in gray

Patient-specific geometry was derived from contrast-enhanced axial CT scans. Due to pronounced metal-induced artifacts from the LVAD device, automated segmentation using neural networks was unreliable. As a result, semi-automatic and manual segmentation was performed in 3D Slicer (Slicer 2024) to delineate the blood volumes of the left ventricle (LV), left atrium (LA), aorta, coronary arteries, and both the inflow and outflow cannulae of the LVAD (Fig. 2b). The images were segmented at the end diastole timeframe. The mitral valve opening area during ventricular filling was defined on the mitral annulus plane as an ellipsoidal surface based on measurements extracted from the patient’s echocardiogram data (Fig. 4, yellow ellipse). The left ventricular wall was segmented to compute LV wall volume and estimate myocardial mass. This mass was then used to scale coronary blood flow, assuming a perfusion rate of 80 mL/min per 100 g of myocardial tissue, consistent with prior work (Barral and Croibier 2011).

A tetrahedral volume mesh with boundary layers was generated using SimModeler meshing tools (Inc 2024). The resulting mesh had an average edge length of 1.0 mm, a refined mesh size of 0.5 mm at the valves, and refined boundary layers featuring a minimum edge length of 0.15 mm. A discontinuous mesh was constructed for the pressure field to allow for discontinuities across closed valves. The dimensions of the mitral valve ellipse are defined by the measurements from the patient’s echocardiogram (Fig. 2b).

**Fig. 3:**
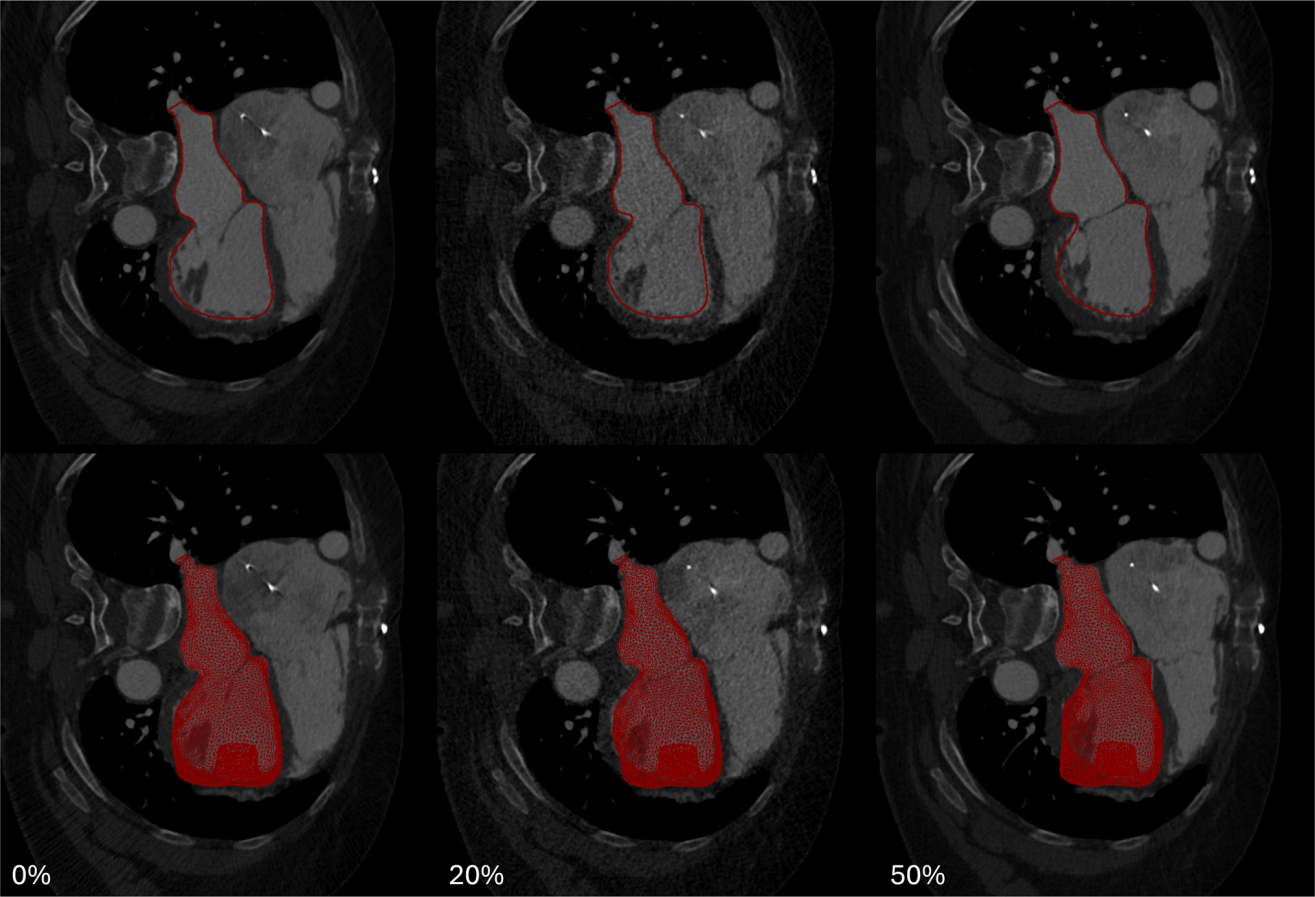
Motion tracking in Eidolon showing the surface on a 2D plane (top row) and the 3D mesh (bottom row) at the 0%, 20%, and 50% of the way through the cardiac cycle

To capture surface deformation of the 3D model, motion was estimated from time-resolved images using the Image Registration Toolkit (IRTK), which computes deformation fields based on non-rigid registration  (Chandrashekara et al. 2024; Shi et al. 2012). The displacement fields are applied to the 3D mesh and used to generate a sequence of surface meshes matching each temporal phase of the CT acquisition (Fig.  3). This process was performed in the biomedical visualization framework Eidolon  (Kerfoot et al. 2016). Temporal interpolation across these states yielded displacement data, which was then differentiated in time to obtain the velocity of the domain boundaries ($\hat{\textbf{v}}_D$).

### Zero-dimensional modeling and model personalization

A zero-dimensional (0D) lumped parameter model was developed to represent the heart chambers, systemic, pulmonary, and coronary circulations, as well as the left ventricular assist device (LVAD) circulation, enabling comprehensive simulation of the cardiovascular system (Fig. 2d). Here, we use the multicompartment 0D circulatory model developed by Hirschvogel and colleagues (Hirschvogel et al. 2017), which consists of 29 state variables and 23 model parameters. More information is found in Appendix B. Briefly, this model capitulates cardiovascular circulation using resistance–inductance–capacitance (RLC) circuit analogues to capture the viscous, inertial, and compliant properties of the vascular compartments. The right heart chambers were represented with time-varying elastance functions to reflect their contractile dynamics. Patient-specific left atrial and left ventricular volumes, extracted from imaging data, were prescribed within the 0D model to enhance model fidelity and consistency with the 3D model. In addition, valve regurgitation was incorporated through regurgitant orifice areas constrained according to clinical severity classifications  (Franz et al. 2021). This reduced-order approach effectively encapsulates the essential hemodynamic features while allowing for computational efficiency.

Personalization of the model was performed by fitting all 23 parameters, including resistances, compliances, elastances, and areas of the valve regurgitant orifices, to available patient-specific data using a nonlinear least squares optimization framework (Fig. 2d). This was done by minimizing the following objective function:

1 $$\begin{aligned} \mathcal {J}(\boldsymbol{\theta }) = \sum _{i=1}^N \frac{1}{2}\left( \frac{\hat{y}_i - y_i}{y_i}\right) ^2, \end{aligned}$$

where $\boldsymbol{\theta }$ represents the vector of 0D model parameters, $\hat{y}_i$ denotes model-predicted values, and $y_i$ corresponds to the patient data. Here, N=20 represents the total number of patient-specific data points used for fitting, with each term in the summation corresponding to the normalized squared error between a model-predicted value and its corresponding clinical measurement. The patient data used and the results of this fitting process can be found in Bonini et al. (2025). Optimization was performed using a hybrid approach that combined gradient descent and the Levenberg–Marquardt algorithm to robustly handle nonlinearities and enhance convergence.

**Fig. 4:**
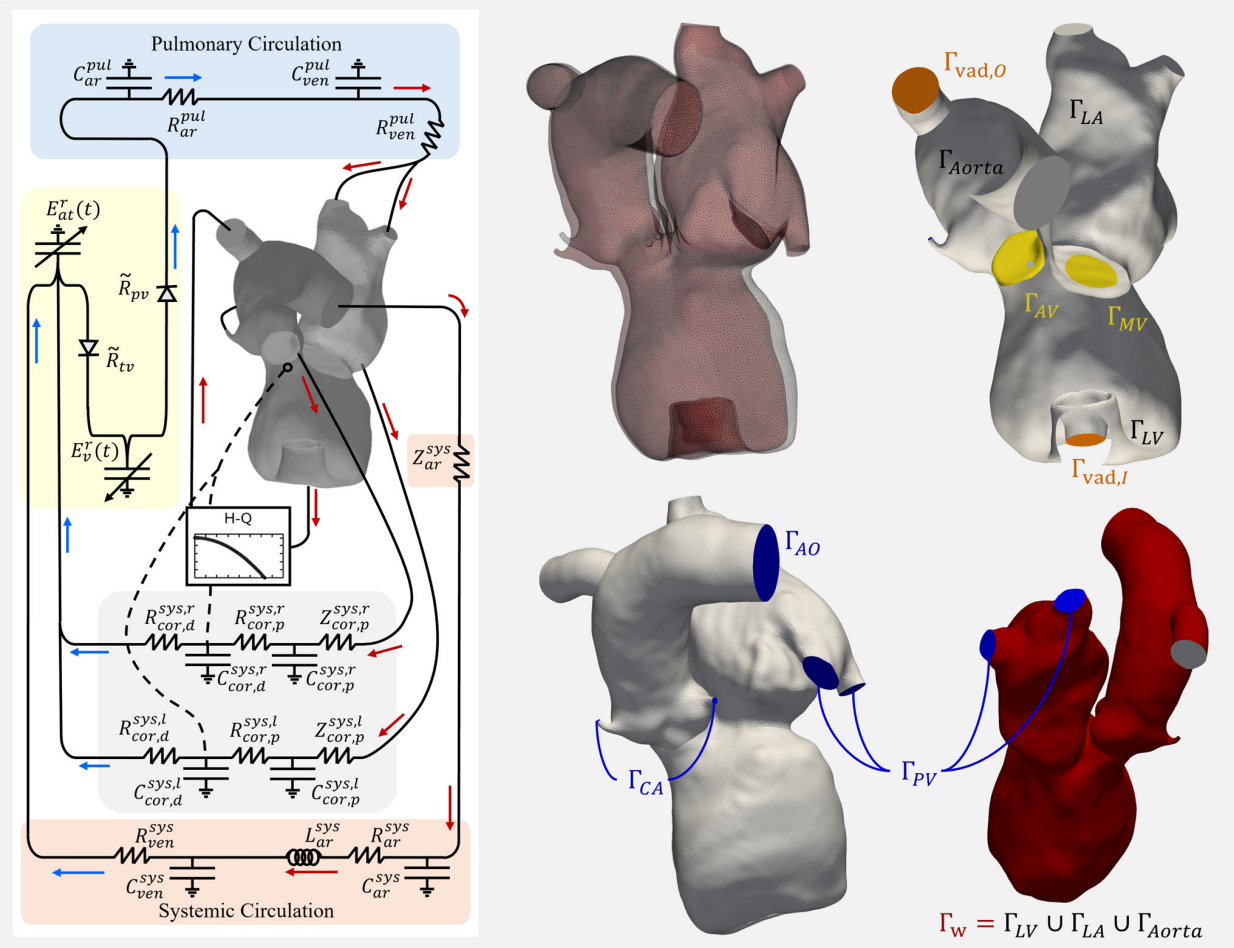
(Left) schematic of the 3D–0D fully coupled, closed-loop model. The systemic system is shaded in orange, the right heart is shaded in yellow, and the pulmonary circulation is shaded in light blue. The coronary circulation is shaded in light gray and incorporates the pressure of the LV and the right atrium. (Right) An overlay of the surface mesh at end diastole (gray) and end systole (red) is shown. The boundaries are shaded by color. The blue faces are coupled to the 0D model: aortic root outflow face ($\Gamma _{AO}$), pulmonary veins ($\Gamma _{PV}$), and coronary arteries ($\Gamma _{CA}$). The known domain motion ($\hat{\textbf{v}}$) is prescribed with a Dirichlet condition on $\Gamma _W$ (red). The mitral ($\Gamma _{MV}$) and aortic valves ($\Gamma _{AV}$) are modeled as surfaces shown in yellow. The 3D model is coupled to the LVAD inflow and outflow cannula at the orange boundaries ($\Gamma _{\text {vad},I},\Gamma _{\text {vad},O}$). *Abbreviations:* *R* or *Z*, resistance; *C*, capacitance; *L*, inductance; *E*, elastance; *pul*, pulmonary; *sys*, systemic; *ven*, veins; *ar*, arteries; *cor*, coronary; *r*, right; *l*, left; *p*, proximal; *d*, distal; *v*, ventricle; *at*, atrium; *tv*, tricuspid valve; *pv*, pulmonary valve; *t*, time; LA, left atrium; LV, left ventricle; vol, volume); *AO*, aortic outflow; *PV*, pulmonary veins

The fitting process was constrained by physiological and clinical considerations to ensure realistic model behavior. Specifically, the total blood volume in the 0D model was required to match the patient-specific blood volume estimated using the Nadler formula, which incorporates patient height, weight, and sex (Nadler et al. 1962). This constraint ensured that the volume distribution among the vascular compartments remained physiologically plausible. In addition, the limits on the valve regurgitate orifice areas were informed by echocardiographic assessments of regurgitation severity. These bounds were enforced during optimization to ensure the estimated parameters remained consistent with the patient’s clinical profile. Upon convergence, the optimized 0D parameters provided a patient-specific characterization of global cardiovascular dynamics. Previously, we performed a local sensitivity analysis to assess the robustness of the model and the identifiability of the parameters. This was done by perturbing each parameter by ±10% and evaluating the resulting change in key hemodynamic outputs  (Bonini et al. 2025). This analysis confirmed that the model was well conditioned, with no single parameter exerting excessive influence, thereby supporting the reliability of the parameter estimation process. To integrate the 0D representation with the three-dimensional finite element model, the optimized parameters were used to define the 0D parameter elements and initial conditions within the fully coupled 3D–0D simulation framework (Fig. 4, left).

### Hemodynamic modeling of the assisted left heart

To evaluate the hemodynamics of the left heart, we couple the arbitrary Lagrangian–Eulerian (ALE) Navier–Stokes equations to 0D circulatory and LVAD pump models and pressure/flow dependent valve conditions. To this end, we considered the time dependent physical domain $\Omega (t) \subset \mathbb {R}^3 \times \left[ 0,T\right]$ with the boundary of the fluid problem being, $\Gamma (t)$ (see Fig. 4, right). We also define a static reference domain $\Omega _0 \subset \mathbb {R}^3$ which can be bijectively mapped to $\Omega (t)$, for all $t\in \left[ 0,T\right]$, i.e.

$$\begin{aligned} \textbf{x}= \boldsymbol{\chi }(\textbf{X},t), \quad \text { for a unique pair } \quad \textbf{x}\in \Omega (t), \quad \textbf{X}\in \Omega _0. \end{aligned}$$

Here, $\boldsymbol{\chi }$ defines the spatial mapping of $\Omega _0$ over time, enabling definition fields that can be defined on either reference or physical domains, interchangeably (Donea et al. 2004). This also enables examination of gradients $\nabla _{0}$ and $\nabla$ over either reference and physical domains. These definitions can be related by the deformation gradient of the mapping, $\textbf{F}_\chi = \nabla _0 \boldsymbol{\chi }$ (e.g. for a vector $\textbf{w}$, $\nabla \cdot \textbf{w} = \textbf{F}_\chi ^{-T}: \nabla _0 \textbf{w}$ and $\nabla \textbf{w} = \nabla _0 \textbf{w} \textbf{F}_\chi ^{-1}$). The formation also enables the definition of the ALE time derivative that can be related to the classic Eulerian derivative, i.e.

$$\begin{aligned} &  \partial _t \textbf{w} (\textbf{X},t) = \lim _{\varepsilon \rightarrow 0} \frac{\textbf{w} (\textbf{X},t+\varepsilon ) - \textbf{w} (\textbf{X},t)}{\varepsilon }, \nonumber \\ \quad &  \partial _t \textbf{w} = \frac{\partial \textbf{w}}{\partial t} + \hat{\textbf{v}}\cdot \nabla \textbf{w}, \nonumber \\ &  \quad \text {where} \quad \hat{\textbf{v}}= \partial _t \boldsymbol{\chi }. \end{aligned}$$

Here, the mapping $\boldsymbol{\chi }$ is defined by boundary motion extracted from dynamic CT, with the internal domain motion found through the solution of an arbitrary partial differential equation (Balmus et al. 2020).

To simulate full-cycle LVAD hemodynamics, in addition to the blood velocity ($$\textbf{v}$$) and pressure (*p*), we also seek to find the domain velocity ($$ \hat{\textbf{v}}$$), flow through the LVAD ($$ Q_{\text {vad}}$$), pressure ($$ \lambda _\text {vad}$$) at the LVAD cannular inflow, pressures at all 0D connected boundaries ($$ \lambda _j$$), 0D model variables ($$ \textbf{y} $$), mean chamber pressures ($$P_{LV} $$, $$P_{LA}$$, and $$P_{AO}$$), and valvular flows ($$ Q_{MV}$$ and $$ Q_{AV}$$) that satisfy the strong form system of equations (at any time $$ t \in [0,T]$$):

2 $$\begin{aligned}&\rho \partial _t \textbf{v}+ \rho (\textbf{v}- \hat{\textbf{v}}) \cdot \nabla \textbf{v}- \nabla \cdot \boldsymbol{\sigma } + \gamma _v \Phi _{v} (\textbf{v} - \hat{\textbf{v}}) = \boldsymbol{0}, \quad \text {on } \Omega (t), \end{aligned}$$

3 $$\begin{aligned}&\nabla \cdot \textbf{v}= 0, \quad \text {on } \Omega (t), \end{aligned}$$

4 $$\begin{aligned}&\textbf{v}= \hat{\textbf{v}}, \quad \text {on } \Gamma _{W}(t), \end{aligned}$$

5 $$\begin{aligned}&(\textbf{v}- \hat{\textbf{v}}) + (Q_{\text {vad}} / A_{\text {vad},O}) \textbf{n} = \boldsymbol{0}, \quad \text {on } \Gamma _{\text {vad},O} (t), \end{aligned}$$

6 $$\begin{aligned}&\mathcal {F} (\textbf{v}-\hat{\textbf{v}}, \Gamma _{0D,j}(t)) - \textbf{y} \cdot \hat{\textbf{e}}_{Q,j} =0, \quad j \in \{ 1,...,{N^{0D}_b} \}, \end{aligned}$$

7 $$\begin{aligned}&\boldsymbol{\sigma } \cdot {\textbf {n}} + \lambda _j {\textbf {n}} = \boldsymbol{0}, \quad \text {on } \Gamma _{0D,j}(t), \; j \in \{1,...,{N^{0D}_b} \}, \end{aligned}$$

8 $$\begin{aligned}&\boldsymbol{\sigma } \cdot {\textbf {n}} + \lambda _\text {vad} {\textbf {n}} = \boldsymbol{0}, \quad \text {on } \Gamma _{\text {vad},I}(t), \end{aligned}$$

9 $$\begin{aligned}&\nabla _0 \cdot E\left( \textbf{D}_0 \hat{\textbf{v}}+ \kappa (\nabla \cdot \hat{\textbf{v}}) \textbf{I} \right) =\textbf{0}, \quad \text {on } \Omega _0, \end{aligned}$$

10 $$\begin{aligned}&\hat{\textbf{v}}=\hat{\textbf{v}}_D, \quad \text {on } \Gamma _0, \end{aligned}$$

11 $$\begin{aligned}&\textbf{M} \frac{\partial \textbf{y}}{\partial t} - \textbf{f}(t,\textbf{y}) =\textbf{0}, \end{aligned}$$

12 $$\begin{aligned}&\textbf{y} \cdot \hat{\textbf{e}}_{p,j} - \lambda _j =0,\quad j \in \{1,...,{N^{0D}_b}\}, \end{aligned}$$

13 $$\begin{aligned}&[P_{AO} - P_{LV}] - HQ (Q_{\text {vad}}) = 0, \end{aligned}$$

14 $$\begin{aligned}&Q_{\text {vad}} - \mathcal {F}(\textbf{v}- \hat{\textbf{v}}, \Gamma _{\text {vad},I}(t) ) = 0, \end{aligned}$$

15 $$\begin{aligned}&Q_{v} - \mathcal {F}(\textbf{v}- \hat{\textbf{v}}, \Gamma _{v}(t) ) = 0, \quad v = \{ \text {MV, AV} \}, \end{aligned}$$

16 $$\begin{aligned}&P_{c} - \bar{\mathcal {F}}(p\textbf{n}, \Gamma _{p,c}(t) ) = 0, \quad c = \{ \text {LV, LA, AO} \}. \end{aligned}$$

Here, (2–3) denote the ALE Navier–Stokes equations, where $\rho = 1.026 g/mL$ and $\mu = 0.004 Pa \cdot s$ are the blood density and viscosity, respectively; $\boldsymbol{\sigma } = 2 \mu \textbf{D} \textbf{v} -p{\textbf {I}}$ is the Cauchy stress tensor; $\gamma _v$ denotes the surface Dirac delta function defined for valve surfaces $\Gamma _v$, $v \in \{ MV, AV \}$; $\Phi _{v}$ denotes penalties to constrain valve opening or closure on mitral ($\Phi _{MV} = \Phi _{MV} (P_{LV}, P_{LA},Q_{MV})$) and aortic ($\Phi _{AV} = \Phi _{AV} (P_{LV}, P_{AO},Q_{AV})$) valves; $\hat{\textbf{v}}_D$ is the boundary velocity found through image tracking; $A_{\text {vad},O} = |\Gamma _{\text {vad},O} |$ is the area of the LVAD cannula flowing out of the LVAD and into the aorta; $\mathcal {F}$ is the normal flux and $\bar{\mathcal {F}}$ the mean flux^1^; $\hat{\textbf{e}}_{Q,j}$ and $\hat{\textbf{e}}_{p,j}$ denote unit vectors that isolate 0D model variables for flow, *Q*, and pressure, *p*, at indicated boundaries *j*; $N^{0D}_b=7$, is the number of boundaries coupling the 3D problem to the 0D lumped parameter model. $\textbf{D}_0$ and $\textbf{D}$ is the symmetric derivative in the ALE reference and physical spaces, respectively. Equations (9–10) denote the ALE grid deformation problem, where $E \in L^2 (\Omega _0)$ denotes a spatially varying dynamic modulus, and $\kappa =1$ denotes a scalar that penalizes volumetric change in the deformed grid. A Dirichlet condition is applied to the domain velocity on $\Gamma _0=\Gamma _W \cup \Gamma _{AO} \cup \Gamma _{CA} \cup \Gamma _{PV} \cup \Gamma _{MV} \cup \Gamma _{AV} \cup \Gamma _{vad,I} \cup \Gamma _{vad,O}$. The 0D lumped parameter model is denoted by Eq. 11, allowing coupling between hemodynamic results and the rest of the cardiovascular system (Eq. 12). Defining the adaptive flow through the LVAD is governed by Eq. (13), where *HQ* denotes the pump-specific pressure / flow curves. Equations (14–16) denote a series of computed pressure / flow quantities that are used to govern valve resistances ($\Phi _v$) and VAD pump flows.

### Numerical solution to simulate the assisted left heart Hemodynamics

In this section, we review the numerical approach to solving the model system of Eqs. (2–16), providing specific details for implementation. Using the image-derived motion of the left heart to drive the motion of the atria, ventricle, and aorta, we first solve the domain motion problem to solve for mesh deformation throughout the cardiac cycle. The dynamic grid velocity $\hat{\textbf{v}}$ and the personalized 0D model were integrated to simulate the hemodynamics of the assisted left heart.

#### Spatiotemporal discretization

A standard FEM discretized form is employed, with monolithic integration of computed quantities to enable efficient simulation. The domain was discretized into tetrahedral elements, $\mathcal {T}^h = \{ e_n \}^{N}_{n=1}$. The dynamics were broken down into equidistant timesteps $0 = t_0< t_1< \ldots < t_{N_t} = T$ (with $t^n - t^{n-1} = \Delta _t$). Here, $N_t$ is the total number of timesteps. Velocity, grid velocity, and pressure were all solved using piecewise linear elements, using the respective function spaces,

$$\begin{aligned} \boldsymbol{\mathcal {V}}^h = [S(\mathcal {T}^h)]^3, \quad \boldsymbol{\mathcal {V}}^h_g = [S(\mathcal {T}^h)]^3, \quad \mathcal {W}^h = S_D(\mathcal {T}^h), \end{aligned}$$

where

$$\begin{aligned} S(\mathcal {T}^h) = \left\{ f:\Omega _0^h \rightarrow \mathbb {R}~|~ f \in C^0(\Omega _0^h) \text { and } f|_e\in \mathbb {P}^1(e)~\forall e\in \mathcal {T}^h \right\} . \end{aligned}$$

*S* enforces continuity across element boundaries, meaning that the velocity and grid displacement fields are continuous functions. In contrast, $S_D$, the function space for pressure, allows discontinuities across valve planes, allowing for large differences in pressure across closed valve regions. This is defined as:

$$\begin{aligned} S_D(\mathcal {T}^h) = \left\{ f:\Omega _0^h \rightarrow \mathbb {R}~|~ f \in C^* \text { and } f|_e\in \mathbb {P}^1(e)~\forall e\in \mathcal {T}^h \right\} , \end{aligned}$$

where

$$\begin{aligned} C^* = C^0(\Omega _{0,LV}^h) \cup C^0(\Omega _{0,LA}^h) \cup C^0(\Omega _{0,AO}^h). \end{aligned}$$

#### Numerical solution of the domain motion problem

Prior to simulating the fully coupled 3D–0D VAD system, we first find the motion and velocity of the grid over time. In this case, at each time point $$ t^n$$, we seek to find the domain velocity $$ \hat{\textbf{v}}^{h,n} \in \boldsymbol{\mathcal {V}}_{g,D}^h $$ that satisfies

17 $$\begin{aligned} &  \int _{\Omega _0^h} E \textbf{D}_0 \hat{\textbf{v}}^{h,n}: \textbf{D}_0 \mathbf {\delta }\hat{\textbf{v}}^h + \kappa ([\textbf{F}_g^{h,n-1}]^{-T}:\nonumber \\ &  \nabla _0 \hat{\textbf{v}}^{h,n} ) (\nabla _0 \cdot \mathbf {\delta }\hat{\textbf{v}}^h) dV = 0, \quad \forall \mathbf {\delta }\hat{\textbf{v}}^h \in \boldsymbol{\mathcal {V}}_{g,0}^h, \end{aligned}$$

where

$$\begin{aligned} &  \boldsymbol{\mathcal {V}}_{g,D}^{h,n} = \{ \textbf{w}^h \in \boldsymbol{\mathcal {V}}_g^h \mid \textbf{w}^h = \hat{\textbf{v}}_D \text { on } \Gamma _0 \}, \quad \nonumber \\ &  \boldsymbol{\mathcal {V}}_{g,0}^{h} = \{ \textbf{w}^h \in \boldsymbol{\mathcal {V}}_g^h \mid \textbf{w}^h = \boldsymbol{0} \text { on } \Gamma _0 \}, \end{aligned}$$

denote the solution spaces for the Dirichlet and homogeneous zero Dirichlet problems. The first term provides a linear elastic propagation of the grid motion, with the modulus *E* is computed as a piecewise constant field over each element, with $$ E = 1 / Q(e)^4$$ for each element $$ e \in \mathcal {T}^h$$ in the physical space. Here *Q*(*e*) denotes the element quality, measured as three times the inradius over the circumradius of *e*. This adaptive stiffness allows for propagation of the domain deformation and limits deterioration of element quality in the mesh (Balmus et al. 2020). In addition, we also add a term to weakly penalize motion that is non-divergence free, which helps to further propagate boundary deformations. We note that here we exploit the *arbitrary* choice of the grid motion problem and, to avoid making the system nonlinear, we pose the divergence on the deformed grid at the previous time step.

With the solution to the domain velocity, we may update the physical coordinates of the domain and compute the grid deformation gradient,

18 $$\begin{aligned} \textbf{x}^{h,n} = \textbf{x}^{h,n-1} + \Delta _t \hat{\textbf{v}}^{h,n} \end{aligned}$$

which can be used to solve the remaining 3D–0D system.

#### Numerical solution of the 3D–0D hemodynamic system

To solve the assisted left heart problem in Eqs. (2–8, 11–16), we define the weak form finite element system in Eq. 19. To review each component of the model, we split the weak form into six separate operators which provide details of the fluid mechanical system ($$ \boldsymbol{R}_f$$), 0D lumped parameter model ($$\boldsymbol{R}_{0D}$$) with $$N^{0D}_e$$, 3D–0D coupling conditions ($$\boldsymbol{R}_{3D0D}$$), mitral and aortic valve control ($$\boldsymbol{R}_{\text {vlv}}$$), the LVAD model conditions ($$\boldsymbol{R}_{\text {vad}}$$), and the quantification of mean values used to regulate terms in the model ($$\boldsymbol{R}_U$$). In this case, at each time point $$ t^n$$, we seek to find

$$\begin{aligned} &  \textbf{v}^{h,n} \in \boldsymbol{\mathcal {V}}_{D}^{h,n}, \quad p^{h,n} \in \mathcal {W}^h, \quad \textbf{y}^n \in \mathbb {R}^{N^{0D}_e},\\ &  \lambda _j^{n}, \lambda _\text {vad}^n, P_c^n, Q_v^n \in \mathbb {R}, \end{aligned}$$

with $j \in \{1,\ldots N^{0D}_b\}$, $c \in \{LV, LA, AO \}$, and $v \in \{MV, AV, \text {vad} \}$ that satisfies

19 $$\begin{aligned}&\boldsymbol{R}_f^n ( \textbf{v}^{h}, p^h; \mathbf {\delta }\textbf{v}^h, \delta p^h) \; \; \boldsymbol{R}_{0D}^n (\textbf{y}, \{ \lambda _j \} ; \delta \textbf{y})\nonumber \\&\quad +\boldsymbol{R}_{3D0D}^n (\textbf{v}^h , \textbf{y}, \{ \lambda _j \} ; \mathbf {\delta }\textbf{v}^h, \{ \delta \lambda _j \}) \; \; \boldsymbol{R}_{\text {vlv}}^n (\textbf{v}^h, \{ P_c \}, \{ Q_v \}; \delta \textbf{v}^h) \nonumber \\&\quad + \boldsymbol{R}_{\text {vad}}^n (\textbf{v}^h, \lambda _\text {vad}, P_{\text {AO}}; \mathbf {\delta }\textbf{v}^h, \delta \lambda _\text {vad} )\nonumber \\&\quad +\boldsymbol{R}_U^n (\textbf{v}^h, p^h, \{ P_c \}, \{ Q_v \} ; \{ \delta P_c\}, \{ \delta Q_v \} ) \; = \;0, \end{aligned}$$

for all $\mathbf {\delta }\textbf{v}^{h} \in \boldsymbol{\mathcal {V}}_{0}^h$, $\delta p \in \mathcal {W}^h$, $\delta \textbf{y} \in \mathbb {R}^{N^{0D}_e}$, and $\delta \lambda _j, \delta \lambda _\text {vad}, \delta P_c, \delta Q_v \in \mathbb {R}$. Here $\boldsymbol{\mathcal {V}}_{D}^{h,n}$ and $\boldsymbol{\mathcal {V}}_{0}^h$ denote the solution spaces for the Dirichlet and homogeneous zero Dirichlet problems at time $t_n$. In the remainder of this section, we review each operator independently.

**Stabilized ALE Navier–Stokes (**$\boldsymbol{R}_f$**).** To model the fluid mechanics, we used a standardized stabilized finite element approach, allowing for equal-order discretization of pressure and velocity ($\mathbb {P}^1-\mathbb {P}^1$) elements (Hoffman and Johnson 2006; Hessenthaler et al. 2017). With these formulations, we express the weak form operator,

$$\begin{aligned}&\boldsymbol{R}_f^n ( \textbf{v}^h , p^h ; \mathbf {\delta }\textbf{v}^h, \delta p^h)\\&\quad := \int _{\Omega ^{h,n}} \rho \left[ \frac{\textbf{v}^{h,n} - \textbf{v}^{h,n-1}}{\Delta _t}\right. \\&\qquad \left. + (\textbf{v}^{h,\theta } - \hat{\textbf{v}}^{h,\theta }) \cdot \nabla \textbf{v}^{h,\theta } \right] \cdot \mathbf {\delta }\textbf{v}^h \\&\qquad + 2 \mu \textbf{D} \textbf{v}^{h,\theta } : \textbf{D} \mathbf {\delta }\textbf{v}^h~dV\\&\qquad +\int _{\Omega ^{h,n}}\delta p^h \nabla \cdot \textbf{v}^{h,\theta } + p^{h,\theta } \nabla \cdot \mathbf {\delta }\textbf{v}^h\\&\qquad +\delta _1 \left[ (\textbf{v}^{h,\theta } - \hat{\textbf{v}}^{h,\theta }) \cdot \nabla \textbf{v}^{h,\theta } \right] \cdot \left[ \textbf{v}^{h,\theta } \cdot \nabla \delta \textbf{v}^h \right. \\&\qquad \left. + \nabla \delta p^h \right] ~dV\\&\qquad +\int _{\Omega ^{h,n}}\delta _2 \left( \nabla \cdot \textbf{v}^{h,\theta } \right) \left( \nabla \cdot \mathbf {\delta }\textbf{v}^h \right) \nonumber \\&\qquad +\delta _3 \nabla p^{h,\theta } \cdot \left( \textbf{v}^{h,\theta } \cdot \nabla \mathbf {\delta }\textbf{v}^h +\nabla \delta p^h\right) ~dV \\&\qquad + \int _{\Gamma _O^{h,n}} \frac{\rho {\beta }}{2} \left[ (\textbf{v}^{h,\theta } - \hat{\textbf{v}}^{h,\theta }) \cdot \textbf{n} \right. \\&\qquad \left. - |(\textbf{v}^{h,\theta }-\hat{\textbf{v}}^{h,\theta }) \cdot \textbf{n}| \right] \textbf{v}^{h,n} \cdot \delta \textbf{v}^h~dA, \end{aligned}$$

Here, $\delta _1, \delta _2,$ and $\delta _3$ denote the three cG(1)cG(1) stabilization parameters  (Hessenthaler et al. 2017) defined as:

$$\begin{aligned} \delta _1 = \frac{\rho h}{v_{\max }}, \delta _2 = \rho h v_{\max } \text { and, } \delta _3 = \frac{h}{v_{\max }}, \end{aligned}$$

where *h* is the size of the element and $v_{max}=$1.0 m/s. To prevent backflow divergence on the outflow faces ($\Gamma _O^{h,n}$), the last term in $\boldsymbol{R}_f$ is added as a local stabilization, with $\beta = 0.2$ as recommended in Esmaily Moghadam et al. (2011).

**0D Modeling (**$\boldsymbol{R}_{0D}$**) and 3D–0D Coupling (**$\boldsymbol{R}_{3D0D}$**).** To model the 0D lumped parameter system  (Hirschvogel et al. 2017), the state variables $\textbf{y}$ are found with the following:

$$\begin{aligned} &  \boldsymbol{R}_{0D}^n (\textbf{y}, \{ \lambda _j \}; \delta \textbf{y}):= \Big [ \textbf{M} [\textbf{y}^n - \textbf{y}^{n-1} ] \\ &  \qquad - \Delta _t \left[ \theta \textbf{f}(\textbf{y}^n) +(1-\theta )\textbf{f}(\textbf{y}^{n-1})\right] \Big ] \\ &  \qquad \cdot \textbf{K} \delta \textbf{y} + \sum _{j=1}^{N^{0D}_b} \hat{\textbf{e}}_{p,j} ( \textbf{y}^n \cdot \hat{\textbf{e}}_{p,j} - \lambda _j^n) \end{aligned}$$

Here, $\textbf{M}$ is a matrix that contains rate dependent terms and $\textbf{f}(\textbf{y})$ collects the other contributions. The parameter $\theta =$ 0.5 for a midpoint scheme was used. The matrix $\textbf{K} = \textbf{I} - \sum _{j=1}^{N^{0D}_b} (\hat{\textbf{e}}_{p,j} \otimes \hat{\textbf{e}}_{p,j})$ suppresses test functions associated with coupling pressures at 3D interfaces, so that they can be imposed via the Lagrange multipliers ($\{\lambda _j\}$) in the final constraint term.

The coupling between the lumped parameter model and the 3D fluid model was achieved with the following (Hirschvogel et al. 2024):

$$\begin{aligned} &  \boldsymbol{R}_{3D0D}^n (\textbf{v}^h, \textbf{y}, \{ \lambda _j \}; \mathbf {\delta }\textbf{v}^h, \{ \delta \lambda _j \}) = \sum _{j=1}^{N^{0D}_b} \int _{\Gamma _{0D,j}^{h,n}} \lambda _{j}^{\theta } \textbf{n} \cdot \mathbf {\delta }\textbf{v}^h ~dA\\ + &  \delta \lambda _{j} \left[ \int _{\Gamma _{0D,j}^{h,n}} (\textbf{v}^{h,n} - \hat{\textbf{v}}^{h,n}) \cdot \textbf{n} ~dA -\alpha _j \textbf{y}^n \cdot \hat{\textbf{e}}_{Q,j} \right] , \end{aligned}$$

so that the net flux computed by the 3D model and the 0D model flux ($\textbf{y}\cdot \hat{\textbf{e}}_{Q,j}$) is consistent. Here, $\alpha _j$ accounts for the directionality of the flow ($-1$ if the 0D flux is an inflow to the 3D model and 1 otherwise).

**Modeling Valve Opening and Closure (**$\boldsymbol{R}_{\text {vlv}}$**).** To control flow across the mitral and aortic valve regions, the following spatially and temporally varying penalty term ($\Phi$) was added to the momentum equation on the corresponding valve boundaries (Fig. 4, $\Gamma _{MV,AV}$)  (Bonini et al. 2025):

$$\begin{aligned} &  \boldsymbol{R}_{\text {vlv}}^n (\textbf{v}^h, \{ P_c \}, \{ Q_v \}; \delta \textbf{v}^h) \\  &  \qquad = \sum _v \int _{\Gamma _v^{h,n}} \Phi _v (\textbf{v}^{h,n} - \hat{\textbf{v}}^{h,n}) \cdot \mathbf {\delta }\textbf{v}^h ~dA, \end{aligned}$$

The value of $\Phi$ serves as a localized resistance field: nodes are set to a large constant to impede flow, or zero when the valve is open. The function $\Phi (\textbf{x}, t)$ evolves with time and position to emulate the physiological opening and closing of the valve planes:

$$\begin{aligned}&\Phi _v (\textbf{x}, \{P_c\},\{Q_v\}, t) \\ =&{\left\{ \begin{array}{ll} 0, & \text {if } \left( \frac{{R_{a}(\textbf{x})}}{l_{a,v}G_v}\right) ^2 + \left( \frac{R_{b}(\textbf{x})}{l_{b,v}G_v} \right) ^2 \le G_v(g_v,g_{min}), \\ 10^6, & \text {otherwise}, \end{array}\right. } \end{aligned}$$

Here, $\Phi$ is governed by the valve geometry and its dynamic state. The mitral valve is modeled as an elliptical region, while the aortic valve is represented as a circular opening. For the MV, $l_a$ = 17 mm and $l_b$ = 9 mm represent the semi-axes of the fully open ellipse, derived from short-axis transthoracic echocardiographic imaging (Fig. 2b). The AV, on the other hand, is treated as a circle with $l_a=l_b$ = 13 mm based on measurements from the 3D model valve plane.

The spatial coordinates $R_i(\textbf{x}) = (\textbf{x} - \textbf{x}_\text {com}) \cdot \hat{\textbf{e}}_i$ define the local distance from the center of the valve plane ($\textbf{x}_\text {com}$) along the principal axes $\hat{\textbf{e}}_i$. The opening state $g(t) \in [0,1]$ is determined by a governing ODE, with $g=1$ representing a fully open valve and $g=0$ corresponding to full closure. The valve state *g*(*t*) is governed by the following ODE:

20 $$\begin{aligned} \frac{d g_v}{dt} = \frac{C_1 \, \text {sgn}(\alpha _v)}{2} \left[ 1 + \alpha _v (1 - 2 g_v) \right] , \end{aligned}$$

$$\begin{aligned} \alpha _{\text {MV}}= &  \tanh \left[ C_2 (P_{\text {LV}} - P_{\text {LA}}) + C_3 \left( Q_{\text {MV}} + \frac{d Q_{\text {AV}}}{dt} \right) \right] ,\\ &  \alpha _{\text {AV}} = \tanh \left[ C_2 (P_{\text {AO}} - P_{\text {LV}}) + C_3 \left( Q_{\text {AV}} + \frac{d Q_{\text {AV}}}{dt} \right) \right] , \end{aligned}$$

The dynamics of valve motion are encapsulated in the switching variable $\alpha \in (-1,1)$, which depends on the pressure drop across the valve ($\Delta P_c$), the volumetric flow rate ($Q_v$), and its time derivative. Transvalvular flow $Q_v$ is obtained by computing flux across the valve surface (Eq. 22). Constants were selected to ensure physiological response times: $C_1 = 800$ controls how quickly the valve responds and was chosen so that the valve can fully transition within approximately 1% of the cardiac cycle. $C_2 = 0.001$ $\hbox {Pa}^{-1}$ and $C_3 = 0.0001$ s$\cdot$ $\hbox {mm}^{-3}$ were tuned to achieve stable and smooth valve behavior in simulations.

For patient-specific cases involving regurgitant lesions, such as aortic regurgitation or mitral regurgitation, a minimum value of $g_v$ is enforced to maintain a finite effective orifice area even during closure,

$$\begin{aligned} G_v(g_v,~g_{min})=\max (g_v(\{P_c\},\{Q_v\}, t),~g_{min}). \end{aligned}$$

For this patient, the aortic and mitral valve $g_{min}$ were equal to 0.106 and 0.302 for a regurgitant orifice area of 6 $\hbox {mm}^2$ and 44 $\hbox {mm}^2$, respectively. These regurgitant orifice areas were found during the parameter fitting process (Sect. 2.3).

In this work, we show that this method can solve for the impact of left heart valve repair. To do so, we modeled 1) mitral valve repair, 2) aortic valve repair, and 3) mitral and aortic valve repair. This was done by allowing $g_{min}$ to reach zero, which causes full closure of the respective valves so that there was no regurgitation.

**LVAD model Integration (**$\boldsymbol{R}_{\text {vad}}$**).** To model flow through the LVAD, we couple the three-dimensional blood flow domain to a representation of the pump via a pressure–flow (H–Q) curve (Eq. 21). The pump is characterized by a nonlinear relationship between the pressure difference across the device ($\Delta P_{\text {vad}} = P_{\text {AO}} - \lambda _\text {vad}$) and the flow rate ($Q_{\text {vad}}$), expressed as:

21 $$\begin{aligned} HQ(Q_\text {vad}) := A + B Q_{\text {vad}} + C Q_{\text {vad}}^2 + D Q_{\text {vad}}^3=\Delta P_{\text {vad}}, \end{aligned}$$

where the coefficients *A*, *B*, *C*, and *D* are fitted to the manufacturer-provided H–Q curve (Santiago et al. 2021). The LVAD device modeled for this study was a HeartMate 3, running at 5400 RPM: $A=$14306 Pa, $B=$ −0.05 $\text {Pa}\cdot \text {s}/\text {mm}^3$, $C=$ 4.0$\hbox {e}^{\text {-7}}~\text {Pa}\cdot \text {s}^2/\text {mm}^6$, and $D=$ −8.0$\hbox {e}^{\text {-12}}~\text {Pa}\cdot \text {s}^3/\text {mm}^9$. With this model, the flow rate through the assist device is entirely determined by the pressure field computed in the 3D fluid domain, enabling a fully coupled interaction between the pump model and the 3D hemodynamics. The LVAD model is embedded in the 3D fluid simulation via the following weak form:

$$\begin{aligned}&\boldsymbol{R}_{\text {vad}}^n (\textbf{v}^h, \lambda _\text {vad}, P_{\text {AO}}; \mathbf {\delta }\textbf{v}^h, \delta \lambda _\text {vad} ) \\&\quad = \int _{\Gamma _{\text {vad},I}^{h,n}} \lambda _\text {vad}^{\theta } \textbf{n} \cdot \mathbf {\delta }\textbf{v}^h ~dA \\&\quad + \delta \lambda _\text {vad} \left[ P_{\text {AO}}^n -\lambda _\text {vad}^n - HQ(Q_\text {vad}^n) \right] \\&\quad + \int _{\Gamma _{\text {vad},O}^{h,n}} \zeta \left[ (\textbf{v}^{h,n} - \hat{\textbf{v}}^{h,n}) - \frac{Q_{\text {vad}}^n}{A_{\text {vad},O}} \textbf{n} \right] \cdot \mathbf {\delta }\textbf{v}^h ~dA, \end{aligned}$$

The first term enforces the LVAD inflow boundary condition on $\Gamma _{\text {vad},I}$ via a Lagrange multiplier $\lambda _{\text {vad}}$, which acts as a traction adjusting to satisfy the flow constraint $Q_{\text {vad}} = f_{\text {pump}}(\Delta P_{\text {vad}})$. The second term introduces the test function $\delta \lambda _{\text {vad}}$ to weakly impose this nonlinear constraint defined by the H–Q relationship. The final term applies a penalty-based weak constraint (with penalty coefficient $\zeta = 10^6$) at the outflow surface $\Gamma _{\text {vad},O}$ to enforce a plug-flow velocity profile consistent with the computed flow rate $Q_{\text {vad}}$.

**Quantifying flows and mean pressures (**$\boldsymbol{R}_U$**).** The following mean values are calculated throughout the cardiac cycle to control flow across the valves and through the LVAD: mean pressures in the left ventricle ($P_{LV}$), aorta ($P_{AO}$), and left atrium ($P_{LA}$), and volumetric flow through the aortic valve ($Q_{AV}$), mitral valve ($Q_{MV}$), and LVAD ($Q_{\text {vad}}$). To do this, the following equations are added:

22 $$\begin{aligned}&\boldsymbol{R}_U^n (\textbf{v}^h, p^h, \{ P_c \}, \{ Q_v \} ; \{ \delta P_c\}, \{ \delta Q_v \} ) \nonumber \\&\quad = \sum _v \delta Q_{v} \left[ \int _{\Gamma _v^{h,n}} (\textbf{v}^{h,n} -\hat{\textbf{v}}^{h,n}) \cdot \textbf{n} ~dA - Q_v^n \right] \nonumber \\&+ \; \sum _c \delta P_c \int _{\Gamma _{p,c}^{h,n}} (p^{h,n} - P_c^n) ~dA \end{aligned}$$

where $c \in \{ LV, LA, AO \}$ and $v \in \{MV,AV,\text {vad} \}$. Here, $\Gamma _\text {vad}$ is the surface out of the LV and into the assist device.

#### Numerical implementation and solver details

The following details outline the solver configuration and simulation setup used to perform the fully coupled 3D–0D simulations. We utilized the finite element-based solver, $\boldsymbol{\mathcal {C}}{{\textbf {Heart}}}$, to perform fully coupled 3D–0D computational fluid dynamics (CFD) simulations (Lee et al. 2016). The system was solved using a monolithic, strongly coupled framework, in which all governing equations were simultaneously solved at each Newton iteration to ensure consistency across submodels. The 3D domain was initialized with zero velocity and zero pressure, while the 0D model was initialized using the steady-state solution obtained from the standalone 0D simulation described in Sect. 2.3. A GMRES iterative solver was employed together with a $3\times 3$ block preconditioner, constructed from a block factorization of the monolithic system and incorporating approximations to the corresponding Schur complements (Hirschvogel et al. 2025). An $\hbox {L}_2$ residual convergence tolerance of $10^{-6}$ was used. All computations were run on the Great Lakes computing cluster at the University of Michigan that has a standard partition consisting of 455 computing nodes, each comprising 36 cores (2 x 3.0 GHz Intel Xeon Gold 6154 processors) (University of Michigan yyy). The simulation for this paper utilized 5 nodes and 175 cores. Each cardiac cycle requires approximately 22–24 h of wall-clock time. Across a full cardiac cycle, a total of $\sim$1333 newton iterations were performed. Simulations were advanced for at least three cardiac cycles, and results from the final cycle were used for analysis once a periodic steady state was achieved, defined as less than 5% change in 0D pressures and flow rates between successive cycles.

## Results

In this work, we developed a method to model the hemodynamics in patients with LVADs, capturing their patient-specific 3D blood flow and pressure in the left heart and aorta, and modeling their global hemodynamics. The method also allows for modeling mitral and aortic valve disease and how it interacts with the surrounding fluid chambers (LA, LV, and aorta). The specific patient we modeled had mild mitral regurgitation, mild aortic and pulmonary regurgitation, and moderate tricuspid regurgitation. The methodology, described above, allowed us to model the patient-specific state as well as the case with mitral valve, aortic valve, and mitral and aortic valve repair.

### Patient-specific results

**Fig. 5:**
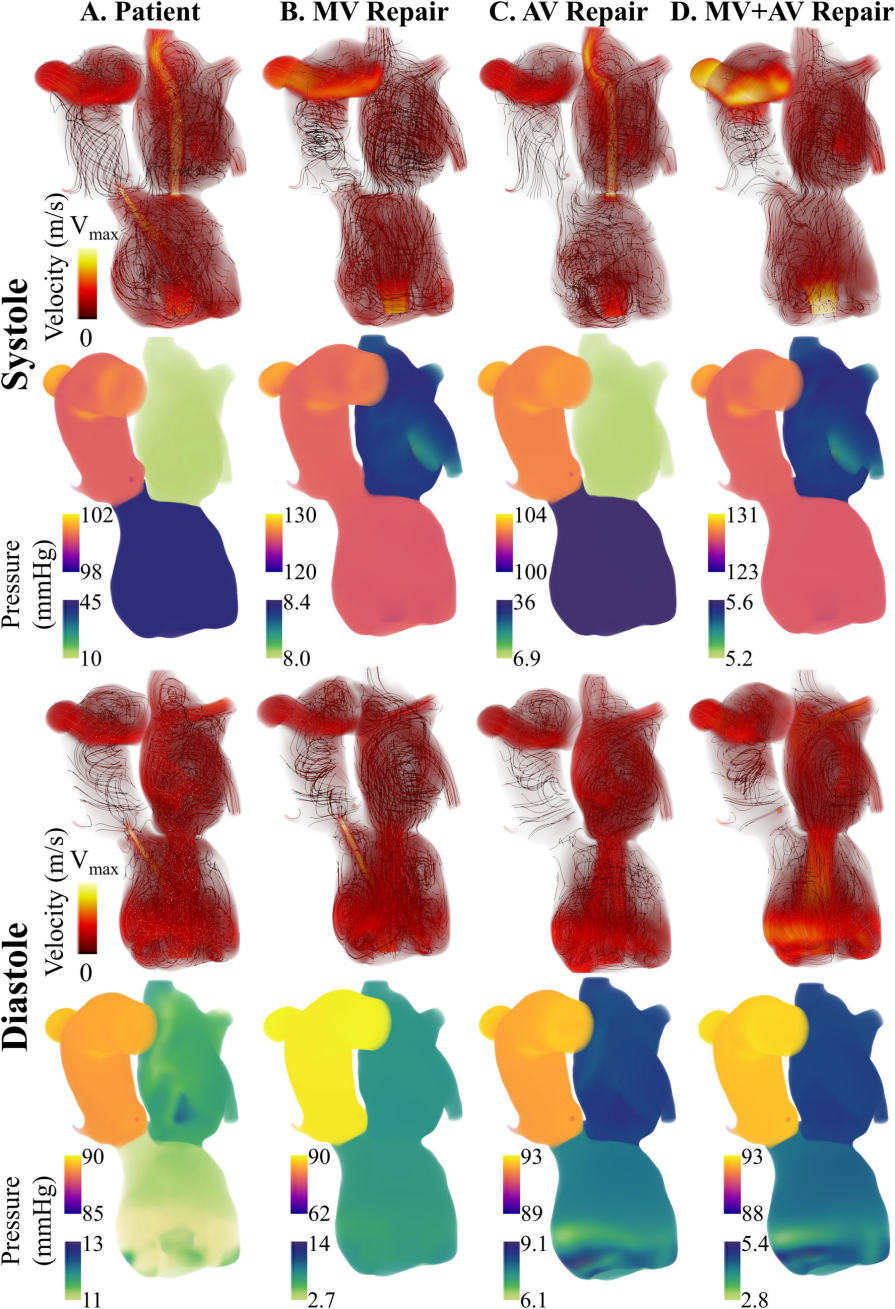
Blood velocity and pressure results at peak systole and end diastole comparing results for the patient-specific case and all cases of valve repair. Note that the pressure range changes for each case. $\hbox {V}_{max}=$1.5 m/s for columns A, B, and C and $\hbox {V}_{max}=$0.9 m/s for column D

**Fig. 6:**
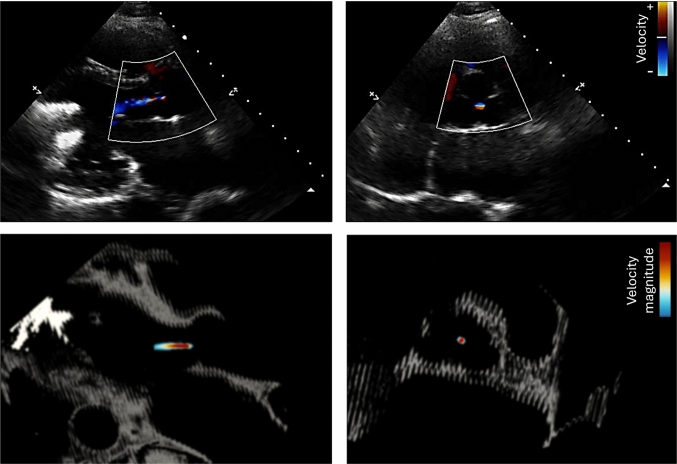
Color Doppler echocardiography images (top row) showing two different views of the aortic valve regurgitant jet. The Doppler flow is colored based on magnitude and direction, with flow moving toward the probe as positive and the flow moving away from the probe as negative. Corresponding computational fluid dynamics (CFD) solutions are shown in the bottom row, aligned to the same views. Here, the flow is colored only by the velocity magnitude. The CFD results are overlaid on the patient’s CT-derived anatomy to visualize the surrounding cardiac structures

Overall, both 0D and 3D–0D models reproduced key physiological targets with acceptable accuracy. The 3D–0D models had an average 8.6% error compared to the patient data. Flow and pressure results from the 3D–0D model are shown in Fig. 5. During systole, a mitral regurgitant jet was observed entering the left atrium and extending toward the pulmonary veins, with a total regurgitant volume of 6.6 mL. The patient’s echocardiogram study reported an early passive filling (E-wave) velocity of 66.0 cm/s. Our model results in an E-wave velocity of 84.6 cm/s. Throughout the cardiac cycle, aortic regurgitation was present, generating a small but persistent retrograde jet through the aortic valve. The total regurgitant volume was 6.5 mL, and the aortic backflow was a small and central jet, consistent with echocardiographic observations (Fig. 6). There was insufficient contraction during systole to open the aortic valve and cause forward flow. Blood flow in the aortic root remained low and non-pulsatile due to aortic valve closure and the constant outflow generated by the LVAD, which provided a mean cardiac output of 3.7 L/min. As a result, the aortic pressure was sustained throughout diastole, in contrast with the typical pressure decay seen in patients with native ventricular ejection. The maximum pressure drop in the aorta over one cardiac cycle was 13.5 mmHg. Despite the LVAD producing an output of 3.7 L/min, the AR caused the forward CO to be 3.16 L/min.

#### Valve modeling

**Fig. 7:**
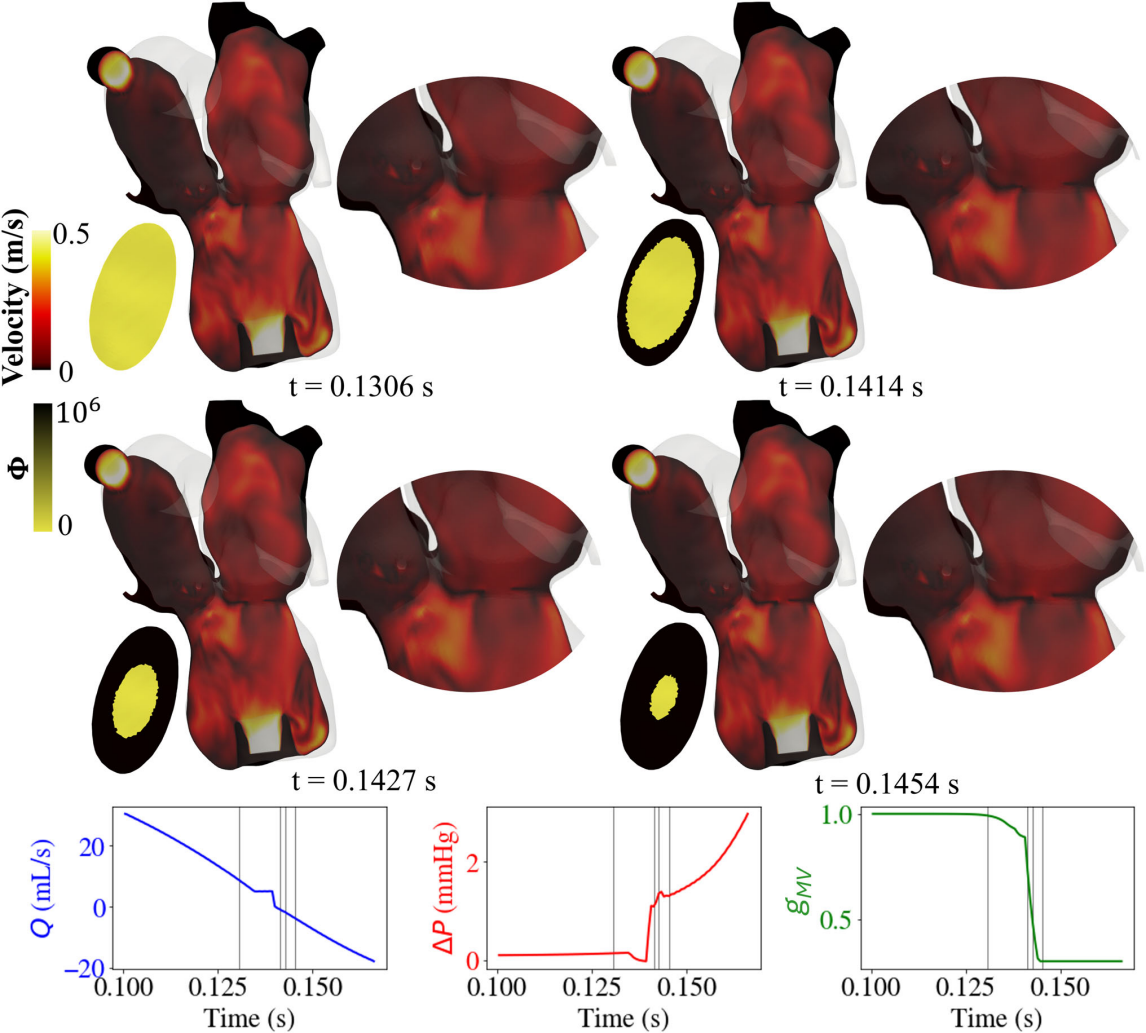
Mitral valve dynamics during closure. The magnitude of velocity in the cut plane of the model is shown for four time steps. The 3D model’s surface is gray and opaque. A zoomed-in view of the mitral valve region is plotted to the right of each model where you can see a black line forms (low-velocity magnitude) as the MV closes and the velocity on those nodes equals the prescribed boundary velocity. The resistance is plotted on the MV plane (left of each model). Here, a high resistance restricting flow is plotted in black, and a low resistance allowing flow is plotted in yellow. The transvalvular volumetric flow (Q), pressure gradient ($\Delta$P), and solution $g_{MV}$ of Eq. 20 are plotted on the bottom. The vertical gray lines correspond with the timings of the figures above

An example of valve dynamics is illustrated with Fig. 7 for the case of mitral valve closure. As the valve transitions toward closure, the velocity field on the cut plane shows the gradual formation of a low-velocity interface at the mitral orifice, consistent with leaflet apposition. However, complete closure is not achieved, as evidenced by residual flow pathways and the persistent yellow region in the resistance map. This incomplete closure is characteristic of regurgitation and is reflected in the transvalvular flow plot (*Q*), which becomes negative as the left ventricle contracts and forces blood into the left atrium. The pressure gradient ($\Delta P$) rises sharply, consistent with forward atrioventricular pressure decoupling, and the valve state variable $g_{MV}$ decreases but does not fully reach zero, indicating partial closure. The transition from fully open to the most closed state allowed by the regurgitant valve occurs over 0.01 s.

### Mitral valve repair

**Fig. 8:**
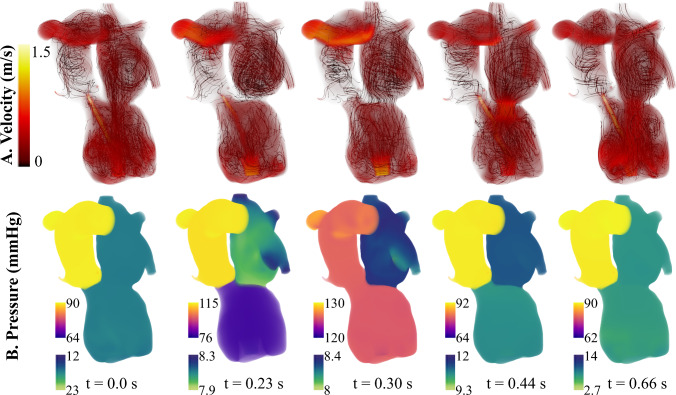
Mitral Valve Repair: left heart and aorta blood flow and pressure over 1 cardiac cycle. Blood flow velocity is shown with volume rendering and streamlines. The pressure is visualized with volume rendering and the pressure range changes for each time point

*In silico* mitral valve repair produced physiologically consistent flow patterns and pressure distributions throughout the cardiac cycle. Regurgitant flow through the mitral valve was substantially reduced, with retrograde velocities in the left atrium markedly diminished compared to the patient-specific case (Fig. 5). The total regurgitant volume over the cardiac cycle decreased from 6.6 mL to 0.13 mL and the forward CO increased from 3.16 L/min to 3.41 L/min. The reduction in mitral regurgitation was associated with lower filling pressure in the left heart and elevated systolic pressures in the left ventricle. This is observed in Fig. 8B, at time t=0.30 s, where the pressure in the LV exceeds that of the aorta, leading to opening of the aortic valve and a forward flow of 0.25 mL/beat. This also leads to pulsatility in the arterial blood pressure that was absent before mitral valve repair. Over the cardiac cycle, the pressure drop in the aortic root was 38.9 mmHg. Mitral valve repair also led to a more physiologically normal LV pressure-volume loop (Fig. 11b, blue). Relative to the pre-repair case, the case of MV repair showed reduced flow disturbance in the left atrium and unloading in the pulmonary venous pressures. This drop in pulmonary pressures and increase in cardiac output led to an 18.9% drop in the peak RV pressure, an 14.2% decrease in the RV end diastolic volume, and an 3.1% increase in RV stroke volume (Fig. 11a, blue).

### Aortic valve repair

**Fig. 9:**
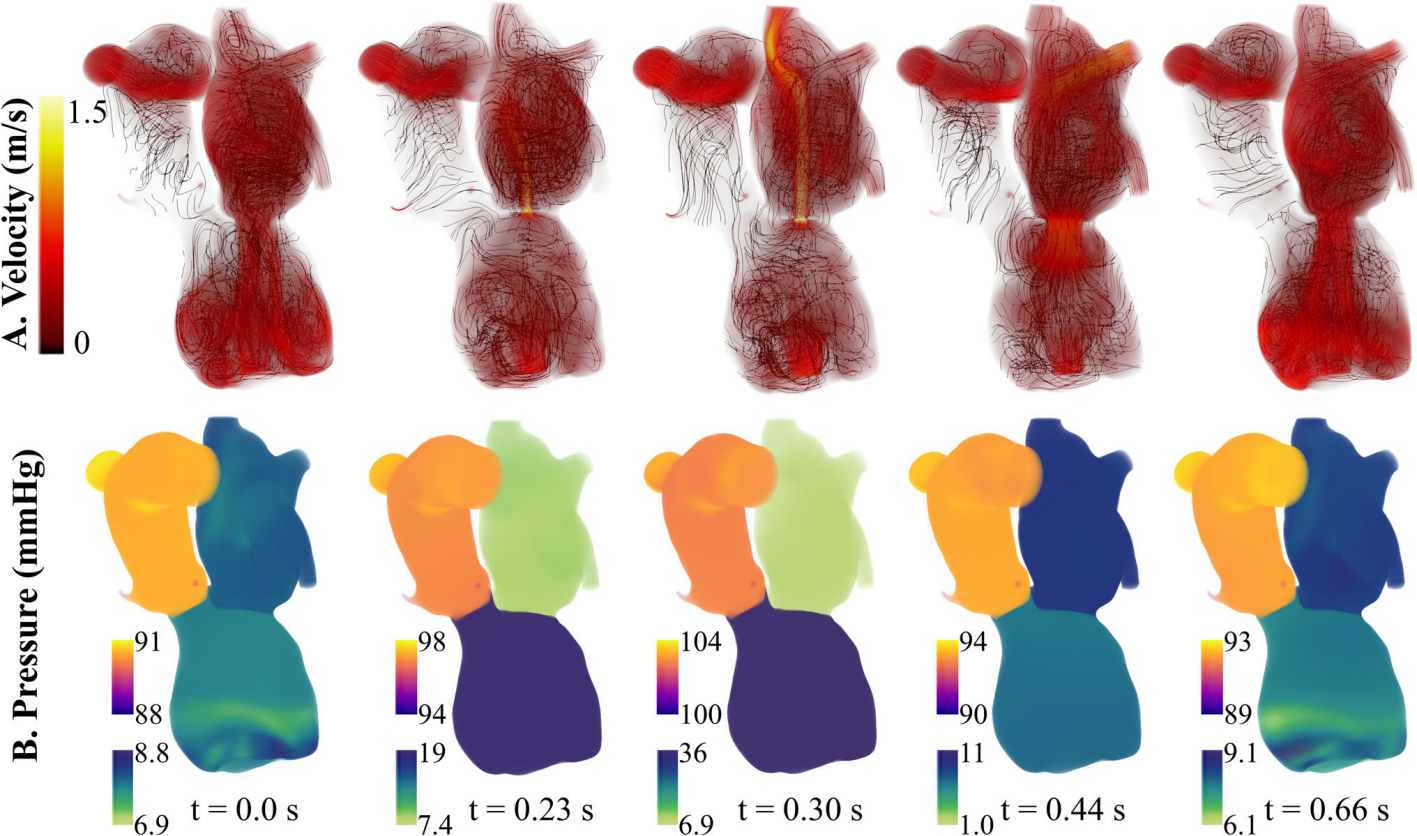
Aortic Valve Repair: left heart and aorta blood flow and pressure over 1 cardiac cycle. Blood flow velocity is shown with volume rendering and streamlines. The pressure is visualized with volume rendering and the pressure range changes for each time point

The repair of the aortic valve removed the small, high-speed jet flowing back into the left ventricle (Fig. 9a). In turn, the velocities in the left ventricle during systole were much lower than the patient-specific case. The AV repair also led to less disturbed flows during ventricular filling, and the vortices commonly associated with diastolic filling reached down into the LV sweeping around the LVAD cannula (Fig. 9a, t=0.66s). Aortic valve repair did not cause opening of the aortic valve and forward flow; however, there was a slightly reduction in the mitral regurgitant volume from 6.6 mL to 6.3 mL compared to the patient-specific case. AV repair also reduced LV filling pressure. The LV end diastolic pressure dropped from 11.4 mmHg in the patient-specific model to 7.9 mmHg. Similar to the case of MV repair, this decreased pulmonary pressures and reduced RV pressure and volume (Fig. 11b). The RV end diastolic volume was 167.0 mL, and the peak systolic blood pressure was 17.7 mmHg. Also, AV repair led to an increase in forward CO (from 3.16 L/min to 3.33 L/min) and therefore an increase in RV stroke volume.

### Aortic valve and mitral valve repair

**Fig. 10:**
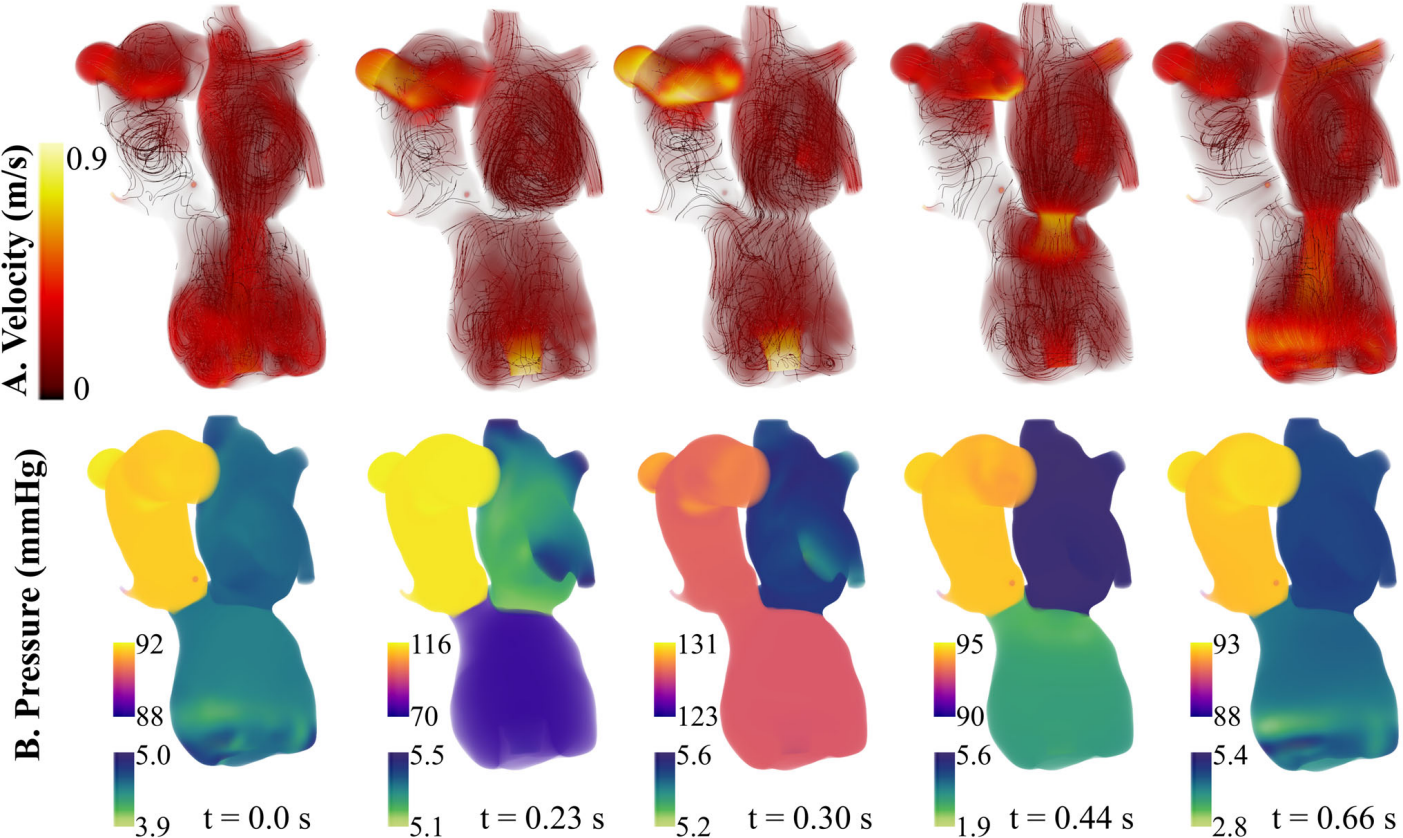
Aortic and Mitral Valve Repair: Left heart and aorta blood flow and pressure over 1 cardiac cycle. Blood flow velocity is shown with volume rendering and streamlines. The pressure is visualized with volume rendering and the pressure range changes for each time point

**Fig. 11:**
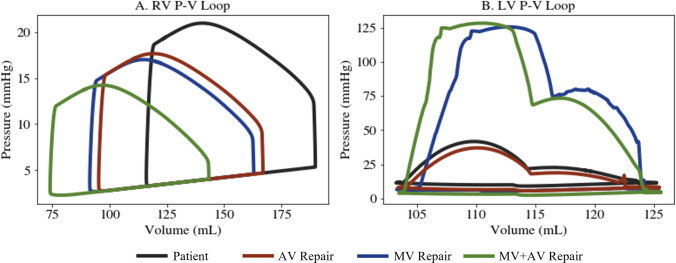
Pressure–Volume Loops for **a** the right ventricle and **b** the left ventricle. The patient-specific case is plotted in black, the case of MV repair in blue, AV repair in red, and mitral and aortic valve repair in green

Together, mitral and aortic valve repair increased forward cardiac output from 3.16 L/min to 3.55 L/min. With valve repair, the flow through the pump matched the forward CO of 3.55 L/min. Overall, the valve repairs reduced the velocities in the left heart and aorta. The peak diastolic filling velocity was 80.6 cm/s. Similar to the case of mitral valve repair, there were an opening of the aortic valve and a forward flow of 0.27 mL/beat. This also caused pulsatile pressures in the aorta, generating a pressure drop of 38.4 mmHg over a cardiac cycle. This is much higher than the patient-specific case and the case of AV repair, where the aortic pressure drop was 15.5 mmHg and 13.7 mmHg, respectively. The change in aortic pressure is observed in Fig. 10b. With both valves repaired, the LV end diastolic pressure was 4.47 mmHg, which is 61% lower than the patient-specific case. This in turn dropped the pulmonary pressures and reduced the RV pressure and volume more than the other cases of valve repair (Fig.  11a). The maximum RV volume was 143.5 mL, and the peak RV blood pressure was 14.22 mmHg.

## Discussion

This work presents a framework for patient-specific computational modeling of LAVD-supported circulation, with the ability to resolve high-resolution 3D flow dynamics while accounting for whole-system physiology through closed-loop coupling to a lumped parameter model. This approach enables detailed analysis of left ventricular and atrial hemodynamics, aortic flow, and the remaining cardiovascular system. This approach is particularly valuable in the context of patients with LVADs, as heart failure is rarely isolated to the LV alone. There are secondary effects, such as right ventricle dysfunction and valvular disease which are often interrelated and clinically significant. Capturing these cross-compartment interactions is essential for understanding patient-specific hemodynamics and guiding optimal LVAD treatment strategies.

In the patient-specific case, both mild mitral regurgitation and mild aortic regurgitation were present. The mitral valve failed to close completely during systole, leading to a regurgitant volume of 6.6 mL and reversal of flow into the pulmonary veins. Simultaneously, the aortic valve remained closed, and continuous retrograde flow from the aorta into the left ventricle contributed an additional 6.5 mL of regurgitant volume. These conditions suppressed native aortic valve opening and resulted in non-pulsatile, low-velocity flow in the aortic root, which has been linked to increased thrombosis risk in patients with LVADs due to stagnant flow and poor washout (Singh 2017). Due to heart failure and the pump, there is minimal deformation compared to a healthy left heart. Our patient-specific model saw good agreement with the available patient data. We observed strong visual agreement between the patient’s echocardiographic assessment of aortic valve regurgitation and the corresponding model predictions (Fig. 6). Similarly, the model reproduced the patient’s E-wave velocity with good accuracy. Some discrepancy between the simulated and measured velocities (84.6 cm/s vs. 66 cm/s) can be attributed to the inherent limitations of echocardiography, particularly when the imaging probe is not perfectly aligned with the direction of blood flow, which leads to systematic underestimation of velocity. Therefore, it is likely that the E-wave velocity for the patient is higher than 66 cm/s and that our model result agrees even more closely with the patient flow. These findings highlight both the consistency of the model with clinical measurements and the complementary role of simulation in providing flow information that may be difficult to capture reliably in practice.

A key advantage of this modeling pipeline is its ability to simulate valvular regurgitation and perform virtual valve repairs. This isolates the impact of valve repair on left heart blood flow and on the remaining cardiovascular system. To do this, we modeled three repair configurations: isolated mitral valve (MV) repair, isolated aortic valve repair, and combined mitral and aortic valve repair. Across all configurations, valve repair reduced pulmonary pressures and left heart filling pressures, indicating more favorable loading conditions. MV repair—either isolated or combined—produced distinct improvements in forward flow, with the combined repair yielding the most physiologic hemodynamic profile overall. In this case, both mitral and aortic regurgitant volumes were nearly eliminated, and the aortic valve opened during systole, restoring forward flow and pressure pulsatility. Notably, isolated AV repair did not result in forward flow through the aortic valve, whereas isolated MV repair did. While this outcome may not be universal across all patients with LVADs, it suggests that MV repair could play a therapeutic role in restoring aortic valve opening and pulsatility—a strategy that is less frequently considered in clinical practice compared to AV repair (Noly et al. 2020).

Repairing the mitral valve resulted in a marked increase in aortic pulsatility compared to pre-repair conditions. This is a physiological characteristic that is largely absent in patients with continuous-flow LVADs  (Witman et al. 2015; Castagna et al. 2017; Balakrishnan and Krishnakumar 2020). Pulsatility is a hemodynamic feature linked to endothelial health, vascular compliance, and long-term vessel integrity (Witman et al. 2015; Roux et al. 2020). In the clinical setting, these parameters are challenging to measure directly, particularly within the ascending aorta, where complex flow patterns and low-velocity regions can develop during LVAD support. Our fully coupled 3D CFD model enables direct quantification of aortic pulsatility and characterization of its spatial distribution. These insights go beyond standard clinical imaging, which typically provides only time-averaged or limited 2D Doppler-derived flow information. By restoring valve competence in silico, we observed more effective left ventricular ejection into the aorta, which in turn re-established the natural systolic–diastolic pressure differential that is often dampened in circulation with LVAD (Imamura et al. 2015; Castagna et al. 2017). This was accompanied by a steeper diastolic pressure decay. From a biomechanical perspective, such normalization of flow may help mitigate the risks that have been reported in patients with non-pulsatile LVADs (Witman et al. 2015; Imamura et al. 2015; Castagna et al. 2017). Importantly, the computational framework allows us to study these effects under controlled conditions, systematically altering valve states while holding all other parameters constant. This capability provides a unique opportunity to disentangle the hemodynamic contributions of valve repair from those of pump speed, patient-specific anatomy, or other comorbidities.

The systemic implications of valve repair were also reflected in the right heart. Across all repair simulations, right atrial pressure and pulmonary artery pressure decreased, consistent with hemodynamic improvements often observed clinically following left heart valve interventions (Rio and Grecu 2018). Reductions in pulmonary pressures suggest a relief of left atrial hypertension and the backward transmission of elevated filling pressures into the pulmonary vasculature. This unloading of the pulmonary circulation decreases right ventricular (RV) afterload, which, together with the increase in overall cardiac output, contributed to the observed improvement in RV ejection fraction—a widely used metric for assessing RV dysfunction. The combined repair scenario demonstrated the largest improvements in these right-sided metrics, underscoring the cumulative benefit of addressing multiple regurgitant lesions. Given that right ventricular failure (RVF) is a leading cause of morbidity and mortality in patients with LVADs (Frankfurter et al. 2020; Bravo et al. 2022), modeling these downstream effects is critical. Our CFD-based framework enables a mechanistic evaluation of how modifications in left-sided valve mechanics propagate through the cardiopulmonary circuit and influence right-sided hemodynamics. By resolving chamber-level flow patterns and pressure distributions, the simulations illustrate how valve repair may alter RV preload and afterload in a manner not easily measured clinically. These results highlight the hemodynamic coupling between the left and right heart and demonstrate the promise of CFD modeling in the clinical evaluation of overall cardiovascular response to surgical or device-based interventions. Further validation will be necessary to assess its ability to characterize RV function directly.

While the proposed framework demonstrates strong potential for guiding treatment decisions, several challenges remain in translating such patient-specific computational modeling into routine clinical practice. First, clinical imaging and patient data are typically acquired only at discrete time points, often during separate visits, so the datasets do not always align temporally. To enable consistent model construction across institutions and patient populations, future efforts must focus on standardizing and homogenizing data acquisition protocols. Furthermore, extreme image artifacts in the CT data further complicate segmentation and boundary condition extraction. Advances in imaging hardware and reconstruction methods—such as photon-counting computed tomography—could reduce artifacts and improve resolution, thereby lowering the uncertainty in geometry. Equally important is the development of streamlined pipelines that couple image processing, model generation, simulation, and clinical visualization into an automated workflow that can operate within the time constraints of clinical decision-making. Addressing these challenges will be critical for integrating high-fidelity computational models into the broader clinical ecosystem, ultimately enabling their use for real-time treatment planning and personalized device optimization.

### Limitations

Several limitations should be considered when interpreting these results. First, the modeling approach relies on image-derived boundary motion rather than fluid–structure interaction. While this enables the capture of patient-specific domain deformation, it limits our ability to simulate changes in myocardial contractility or remodeling in response to altered loading conditions. This is seen in the pressure-volume loops of the left ventricle, where the volume is the same between all cases of valve repair (Fig. 11b). In addition, the pressure–volume loops exhibit a localized pressure excursion during systolic ejection, which arises from abrupt changes in the rate of ventricular volume reduction imposed by the image-derived boundary motion. This behavior reflects the limited temporal resolution of the available imaging data rather than an intrinsic modeling artifact and could be mitigated in future work through higher temporal resolution imaging or improved motion interpolation strategies. Second, the valve surfaces are represented using idealized planes with prescribed orifice areas. While this approach enables dynamic modulation of resistance throughout the cardiac cycle, it does not incorporate detailed leaflet geometry, such as tethering, prolapse, or three-dimensional curvature. Nonetheless, the model successfully captures key hemodynamic behaviors associated with regurgitation and valve closure timing, providing physiologically meaningful insights despite these geometric simplifications. Thirdly, while the lumped parameter model is tuned to match a comprehensive set of clinical measurements, the input data itself are subject to uncertainty. For example, the reported LVAD flow values are known to have an error up to around 1 L/min (Rocchi et al. 2025; Abart et al. 2025). Furthermore, the LVAD is modeled using a static pressure–flow (H–Q) relationship at a fixed operating speed, which neglects dynamic pump response. While this simplification may influence transient pump behavior, other studies suggest that these effects are relatively small at the operating speed used in this paper (Boes et al. 2019); nonetheless, incorporation of dynamic pump models represents an important extension of the present framework. Additionally, the right ventricle is represented using a reduced-order, time-varying elastance model and does not explicitly resolve septal interaction or pericardial constraint. While this is a common approach and has been validated in other works (Comunale et al. 2021), conclusions regarding right-sided behavior are limited to global hemodynamic loading responses rather than detailed ventricular mechanics. Furthermore, the model also does not yet incorporate the cardiovascular system’s dynamic response to pathological conditions and therapeutic interventions. While in this paper valve repair scenarios are simulated by altering valve states while holding all other parameters fixed, we do not expect the cardiovascular system to remain unchanged following real clinical intervention. This controlled approach is used here to isolate causal hemodynamic effects; modeling physiological adaptation and remodeling in response to intervention represent an important direction for future work. Nevertheless, it serves as a powerful tool for investigating patient-specific hemodynamics, providing critical insights that would be challenging to obtain otherwise. Moreover, it establishes a strong foundation for future refinements, including the integration of Fluid-reduced–Solid Interaction (Hirschvogel et al. 2024), to enhance adaptability and broaden its applicability. Finally, the conclusions drawn from this study are based on a single patient case. Given the heterogeneity across patients, additional cases are needed to establish the generalizability of these findings. Future work will therefore focus on expanding the patient cohort and exploring varied physiological conditions, which will be essential for validating the predicted post-intervention trends and establishing the framework’s utility for model-informed clinical decision support.

### Conclusion

This study establishes a comprehensive computational framework that integrates patient-specific imaging and hemodynamic data to investigate the interplay between LVAD support, valve regurgitation, and surgical repair. By combining detailed three-dimensional flow resolution with whole-system physiological coupling, the model captures both localized valve dynamics and global cardiovascular responses. The demonstrated case highlights the framework’s ability to assess the impact of mitral and aortic valve repair on patient-specific hemodynamics, offering a powerful tool for evaluating surgical strategies and improving clinical decision-making.

## Figures and Tables

**Fig. 12:**
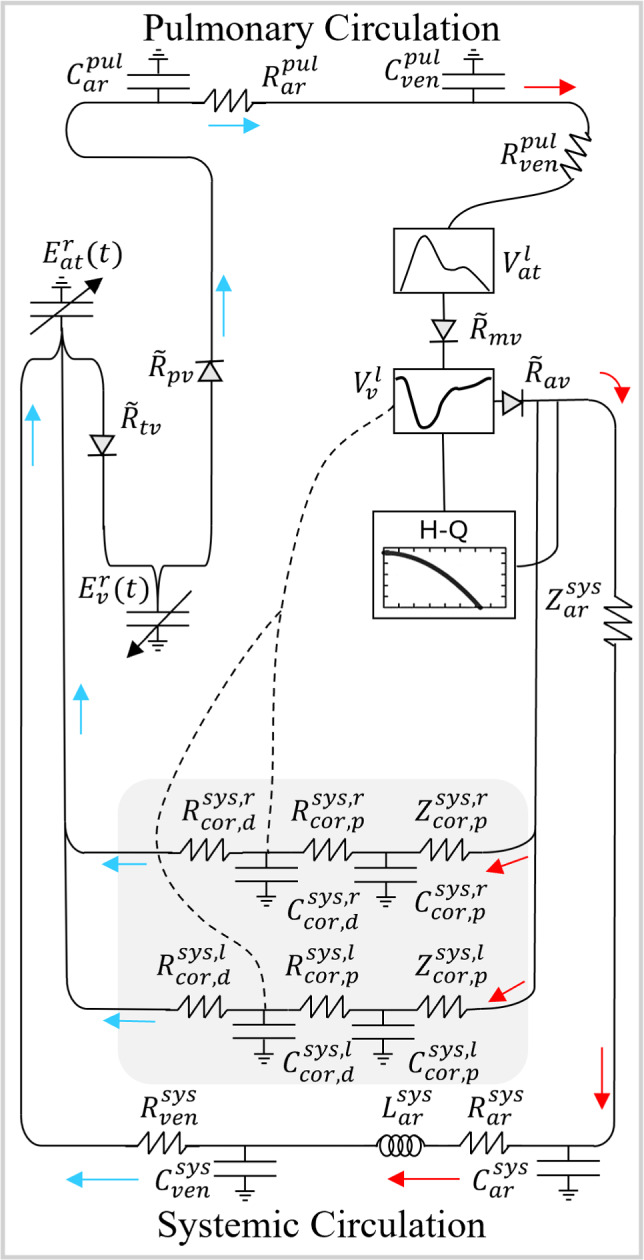
Schematic of the zero-dimensional system. The time-varying LV ($V_{v}^l$) and LA ($V_{at}^l$) volumes are prescribed from patient data. The coronary system is highlighted in gray

## Data Availability

The 3D mesh at each timephase in the cardiac cycle is available via the University of Michigan Deep Blue database: https://doi.org/10.7302/a4yg-e971

## Appendix A: Materials

The following list further describes the data used in this paper: description of the data, the method in which it was processed, and how it was used to build the model. A summary of the quantified data is shown in Table 1.

1. Dynamic contrast-enhanced CT imaging
  - Description: CT imaging was performed using a 64-slice helical scanner (Siemens Somatom Force), yielding spatial resolutions of 0.4886 mm in the sagittal plane, 0.4885 mm in the coronal plane, and 1.25 mm in the axial direction. Scans were acquired during iopamidol contrast administration and used retrospective ECG gating to reconstruct 10 cardiac phases, enhancing temporal resolution.
  - Method: We segmented the frame at end diastole using the software Slicer (Slicer 2024). We used semi-automatic tools, including thresholding, to segment the LV, LA, pulmonary veins, aorta, coronary arteries, RV, RA, LVAD, and LVAD inflow and outflow cannuli. We also segmented the LV myocardium to calculate the LV myocardium volume.
  - Utilization: The segmentation of the LV, LA, pulmonary arteries, aorta, coronary arteries, LVAD, and LVAD cannuli was used to construct the 3D model of the left heart and aorta as shown in Fig. 4. The end-diastolic volume of the RV and the RA was used as data points during the 0D parameter optimization process (Sect. 2.3). The LV myocardium volume was used to calculate the LV myocardium mass. We assumed that the density of the myocardium was 1.055 g/ml (Vinnakota and Bassingthwaighte 2004). The myocardium mass was used to estimate the amount of coronary flow the patient would need. We assumed that the patient would need 80 ml/min per 100 g of tissue (Barral and Croibier 2011).
2. LVAD Information
  - Description: A summary of the LVAD device type, pump speed, and flow rate is reported for the patient.
  - Method: These data were collected from the patient reports in the hospital database.
  - Utilization: The LVAD pump speed was used to define the coefficients of the HQ curve shown in Eq. 21. The LVAD flow rate was used to help fit the 0D parameters as described in Sect. 2.3
3. Right Heart Cardiac Catheterization
  - Description: Right heart cardiac catheterization data was analyzed.
  - Method: Procedural results were summarized in a clinical report. Extracted data included mean and peak right atrial pressures; right ventricular systolic, diastolic, and end-diastolic pressures; mean and maximum pulmonary capillary wedge pressures; systolic and diastolic pulmonary artery pressures; and mean pulmonary artery pressure.
  - Utilization: These data were used to calibrate the parameters of the 0D circulatory model, as described in Sect. 2.3 and in Bonini et al. (2025). We used the pulmonary capillary wedge pressure as a proxy for the LA pressure.
4. Echocardiogram
  - Description: Two-dimensional transthoracic echocardiogram (TTE) images and summary report.
  - Method: Echocardiogram images were visualized and analyzed using Synapse Cardiovascular Client (v6.22). One image set captured mitral valve opening and closing over the cardiac cycle. Images were acquired at a frame rate of 30 frames per second. At the phase of maximal mitral valve opening, the software measurement tool was used to quantify the long- and short-axis dimensions of the open mitral valve orifice.
  - Utilization: The echocardiogram report provided clinical assessments of aortic regurgitation and mitral regurgitation severity. These severity grades were used to determine the corresponding valve regurgitant orifice areas such that the resulting backflow volumes were consistent with the clinically reported severity for each patient. This was done during the 0D optimization process (Sect. 2.3).
5. Medical Records
  - Description: Patient medical record reports were utilized.
  - Method: Data were accessed through the MiChart clinical database. Extracted information included noninvasive systemic systolic and diastolic blood pressures, sex, height, weight, and the aforementioned summarized findings from right heart catheterization and echocardiography reports.
  - Utilization: Noninvasive systemic arterial systolic and diastolic blood pressures were used to optimize parameters in the 0D model. Sex, height, and weight were used to estimate total patient blood volume using the Nadler formula (Nadler et al. 1962). This estimate was then used to constrain the total blood volume in the 0D system during the parameter fitting process.

**Table 1:** Clinical data

Exam	Data	
Dynamic CT imaging	Right Ventricle End Diastolic Volume (mL)	185.2
	Right Atrium End Diastolic Volume (mL)	154.3
	Left Ventricle Wall Volume (mL)	126.6
LVAD Information	Device Type	Heartmate 3
	Pump Speed (rpm)	5400
	Flow Rate (L/min)	3.7
Right Heart Cardiac Catheterization	Systolic Pulmonary Pressure (mmHg)	21.0
	Diastolic Pulmonary Pressure (mmHg)	12.0
	Mean Pulmonary Pressure (mmHg)	15.0
	Right Ventricle Systolic Pressure (mmHg)	20.0
	Right Ventricle Diastolic Pressure (mmHg)	3.0
	Right Ventricle End Diastolic Pressure (mmHg)	5.0
	Mean Right Atria Pressure (mmHg)	5.0
	Right Atria V Wave (mmHg)	8.0
	Right Atria A Wave (mmHg)	6.0
	Mean Pulmonary Capillary Wedge Pressure (mmHg)	12.0
	Pulmonary Capillary Wedge V Wave (mmHg)	13.0
	Pulmonary Capillary Wedge A Wave (mmHg)	12.0
	Heart Rate (bpm)	90
Echocardiogram (TTE)	Mitral Valve Regurgitation	mild
	Aortic Valve Regurgitation	mild
	Tricuspid Valve Regurgitation	moderate
	Pulmonary Valve Regurgitation	mild
	Mitral Valve Orifice Dimensions (mm)	17x9
Medical Records	Systolic Systemic Artery Pressure (mmHg)	94.0
	Diastolic Systemic Artery Pressure (mmHg)	68.0
	Height (inch)	63
	Weight (lb)	130
	Sex	Female

## Appendix B: Zero-dimensional model

The lumped parameter (0D) representation captures the principal elements of the cardiovascular system (Fig. 12). We model the systemic arteries and veins, the coronary system, and the pulmonary arteries and veins. The right atrium and ventricle are modeled with elastance functions. The pressure–volume dynamics of the right atrium and ventricle are expressed as, $p(t) = E(t)\cdot V(t)$, with *p* denoting chamber pressure, *V* the chamber volume, and *E*(*t*) the time-varying elastance. The elastance function is parameterized as $E(y) = (E_{max}-E_{min}) \cdot y(t) + E_{min}$.

**Table 2:** 0D model parameters fit during optimization

Parameter	Value
MV Regurgitant Orifice Area, $A_{o}^{MV}$ ($\hbox {cm}^2$)	0.44
AV Regurgitant Orifice Area, $A_{o}^{AV}$ ($\hbox {cm}^2$)	0.06
TV Regurgitant Orifice Area, $A_{o}^{TV}$ ($\hbox {cm}^2$)	0.59
PV Regurgitant Orifice Area, $A_{o}^{PV}$ ($\hbox {cm}^2$)	0.18
Systemic Artery Impedance, $Z_{\textrm{ar}}^{\textrm{sys}}$ (Pa$\cdot$s/$\hbox {mm}^3$)	5.64e−2
Systemic Artery Capacitance, $C_{\textrm{ar}}^{\textrm{sys}}$ ($\hbox {mm}^3$/Pa$\cdot$s)	86.53
Systemic Artery Resistance, $R_{\textrm{ar}}^{\textrm{sys}}$ (Pa$\cdot$s/$\hbox {mm}^3$)	9.89e−2
Systemic Artery Inductance, $L_{\textrm{ar}}^{\textrm{sys}}$ (Pa$\cdot$ $\hbox {s}^2$/$\hbox {mm}^3$)	0.667e−3
Systemic Vein Capacitance, $C_{\textrm{ven}}^{\textrm{sys}}$ ($\hbox {mm}^3$/Pa$\cdot$s)	494.33
Systemic Vein Resistance, $R_{\textrm{ven}}^{\textrm{sys}}$ (Pa$\cdot$s/$\hbox {mm}^3$)	6.51e−2
Coronary Artery Proximal Impedance, $Z_{\textrm{cor,p}}^{\textrm{sys}}$ (Pa$\cdot$s/$\hbox {mm}^3$)	1.71
Coronary Artery Proximal Capacitance, $C_{\textrm{cor,p}}^{\textrm{sys}}$ ($\hbox {mm}^3$/Pa$\cdot$s)	0.055
Coronary Artery Proximal Resistance, $R_{\textrm{cor,p}}^{\textrm{sys}}$ (Pa$\cdot$s/$\hbox {mm}^3$)	2.12
Coronary Vein Distal Capacitance, $C_{\textrm{cor,d}}^{\textrm{sys}}$ ($\hbox {mm}^3$/Pa$\cdot$s)	0.096
Coronary Vein Distal Resistance, $R_{\textrm{cor,d}}^{\textrm{sys}}$ (Pa$\cdot$s/$\hbox {mm}^3$)	6.48
RA Minimum Elastance, $E_{\textrm{min}}^{\textrm{RA}}$ (Pa/$\hbox {mm}^3$)	2.39e−3
RA Maximum Elastance, $E_{\textrm{max}}^{\textrm{RA}}$ (Pa/$\hbox {mm}^3$)	5.45e−3
RV Minimum Elastance, $E_{\textrm{min}}^{\textrm{RV}}$ (Pa/$\hbox {mm}^3$)	3.76e−3
RV Maximum Elastance, $E_{\textrm{max}}^{\textrm{RV}}$ (Pa/$\hbox {mm}^3$)	2.08e−2
Pulmonary Artery Capacitance, $C_{\textrm{ar}}^{\textrm{pul}}$ ($\hbox {mm}^3$/Pa$\cdot$s)	29.98
Pulmonary Artery Resistance, $R_{\textrm{ar}}^{\textrm{pul}}$ (Pa$\cdot$s/$\hbox {mm}^3$)	9.6e−3
Pulmonary Vein Capacitance, $C_{\textrm{ven}}^{\textrm{pul}}$ ($\hbox {mm}^3$/Pa$\cdot$s)	118.35
Pulmonary Vein Resistance, $R_{\textrm{ven}}^{\textrm{pul}}$ (Pa$\cdot$s/$\hbox {mm}^3$)	0.11e−3

The coronary artery parameters are used for the left (*l*) and right (*r*) coronary arteries

Here *y*(*t*) is the activation function describing contraction over time, and $E_{max}$ and $E_{min}$ represent the upper and lower bounds of chamber elastance. For example, during diastole, $y(t)=0$, for the right ventricle, and only the minimum elastance would be in effect. We define the volumetric curves of the left ventricle and left atrium. The left atrium and left ventricle volumetric fluxes are defined as $Q_{\textrm{at}}^{\ell }:= -\textrm{d}V_{\textrm{at}}^{\ell }/\textrm{d}t$ and $Q_{\textrm{v}}^{\ell }:= -\textrm{d}V_{\textrm{v}}^{\ell }/\textrm{d}t$, respectively.

For this patient, there was regurgitation at all cardiac valves. To model valve disease in the 0D model, the following equation from Franz et al. was used

23 $$\begin{aligned} q(p-p_{\textrm{open}}) = {\left\{ \begin{array}{ll} c A_{\textrm{o}} \sqrt{p-p_{\textrm{open}}}, p < p_{\textrm{open}} \\ \frac{p-p_{\textrm{open}}}{R_{\min }}, p \ge p_{\textrm{open}} \end{array}\right. } \end{aligned}$$

where $p_{open}$ is the downstream pressure, *p* is the pressure in the chamber of interest, $R_{min}=$1$\hbox {e}^{-5}$ Pa$\cdot$s/$\hbox {mm}^3$, c=37.2 $\frac{mm/s}{sqrt(Pa)}$ , and $A_o$ is the regurgitant orifice area (Franz et al. 2021). Circulatory pressure–flow interactions across the entire system are governed by first-order ordinary differential equations (ODEs). The full set of governing relations for the 0D cardiovascular model can therefore be written as:

$$\begin{aligned}&{\textbf {Left heart and Systemic Circulation}}\\&-Q_{\textrm{at}}^{\ell } - \sum \limits _{i=1}^4 + q_{\textrm{v,in}}^{\ell } = 0 \\&\frac{{1}}{\tilde{R}_{\textrm{v,in}}^{\ell }}(p_{\textrm{at}}^{\ell }-p_{\textrm{v}}^{\ell }) - q_{\textrm{v,in}}^{\ell } = 0\\&-Q_{\textrm{v}}^{\ell } - q_{\textrm{v,in}}^{\ell } + q_{\textrm{v,out}}^{\ell } = 0\\&\frac{{1}}{\tilde{R}_{\textrm{v,out}}^{\ell }}(p_{\textrm{v}}^{\ell }-p_{\textrm{ar}}^{\textrm{sys}}) - q_{\textrm{v,out}}^{\ell } = 0\\&A + B Q_{\text {vad}} + C Q_{\text {vad}}^2 + D Q_{\text {vad}}^3=p_{ar}^{sys}-p_v^l\\&-Q_{\textrm{aort}}^{\textrm{sys}} - q_{\textrm{v,out}}^{\ell } + q_{\textrm{ar,p}}^{\textrm{sys}} = 0 \\&Z_{\textrm{ar}}^{\textrm{sys}}\,q_{\textrm{ar,p}}^{\textrm{sys}} - p_{\textrm{ar}}^{\textrm{sys}} + p_{\textrm{ar,d}}^{\textrm{sys}} = 0\\&C_{\textrm{ar}}^{\textrm{sys}} \frac{\textrm{d}p_{\textrm{ar,d}}^{\textrm{sys}}}{\textrm{d}t} - q_{\textrm{ar,p}}^{\textrm{sys}} + q_{\textrm{ar}}^{\textrm{sys}} = 0\\&L_{\textrm{ar}}^{\textrm{sys}} \frac{\textrm{d}q_{\textrm{ar}}^{\textrm{sys}}}{\textrm{d}t} + R_{\textrm{ar}}^{\textrm{sys}}\,q_{\textrm{ar}}^{\textrm{sys}} - p_{\textrm{ar,d}}^{\textrm{sys}} + p_{\textrm{ven}}^{\textrm{sys}} = 0\\&C_{\textrm{ven}}^{\textrm{sys}} \frac{\textrm{d}p_{\textrm{ven}}^{\textrm{sys}}}{\textrm{d}t} - q_{\textrm{ar}}^{\textrm{sys}} + q_{\textrm{ven}}^{\textrm{sys}}\ = 0\\&R_{\textrm{ven}}^{\textrm{sys}}\, q_{\textrm{ven}}^{\textrm{sys}} - p_{\textrm{ven}}^{\textrm{sys}} + p_{\textrm{at}}^{r} = 0\\&{\textbf {Right heart and Pulmonary Circulation}}\\&\frac{\textrm{d}}{\textrm{d}t}(\frac{{p_{\textrm{at}}^{r}}}{E_{\textrm{at}}^{r}}) - q_{\textrm{ven}}^{\textrm{pul}} + q_{\textrm{v,in}}^{r} = 0\\&\frac{{1}}{\tilde{R}_{\textrm{v,in}}^{r}}(p_{\textrm{at}}^{r}-p_{\textrm{v}}^{r}) - q_{\textrm{v,in}}^{r} = 0\\&\frac{\textrm{d}}{\textrm{d}t}(\frac{{p_{\textrm{v}}^{r}}}{E_{\textrm{v}}^{r}}) - q_{\textrm{v,in}}^{r} + q_{\textrm{v,out}}^{r} = 0\\&\frac{{1}}{\tilde{R}_{\textrm{v,out}}^{r}}(p_{\textrm{v}}^{r}-p_{\textrm{ar}}^{\textrm{pul}}) - q_{\textrm{v,out}}^{r} = 0\\&C_{\textrm{ar}}^{\textrm{pul}} \frac{\textrm{d}p_{\textrm{ar}}^{\textrm{pul}}}{\textrm{d}t} - q_{\textrm{v,out}}^{r} + q_{\textrm{ar}}^{\textrm{pul}} = 0\\&R_{\textrm{ar}}^{\textrm{pul}}\,q_{\textrm{ar}}^{\textrm{pul}} - p_{\textrm{ar}}^{\textrm{pul}} +p_{\textrm{ven}}^{\textrm{pul}} = 0\\&C_{\textrm{ven}}^{\textrm{pul}} \frac{\textrm{d}p_{\textrm{ven}}^{\textrm{pul}}}{\textrm{d}t} - q_{\textrm{ar}}^{\textrm{pul}} + \sum \limits _{i=1}^{n_{\textrm{ven}}^{\textrm{pul}}}q_{\textrm{ven},i}^{\textrm{pul}} = 0\\&R_{\textrm{ven},i}^{\textrm{pul}}\, q_{\textrm{ven},i}^{\textrm{pul}}-p_{\textrm{ven}}^{\textrm{pul}}+p_{\textrm{at},i}^{\ell }=0\\ \end{aligned}$$

$$\begin{aligned}&{\textbf {Coronary Circulation}}\\&C_{\textrm{cor,p}}^{\textrm{sys},\ell } \left( \frac{\textrm{d}p_{\textrm{ar}}^{\textrm{sys},\ell }}{\textrm{d}t}-Z_{\textrm{cor,p}}^{\textrm{sys},\ell }\frac{\textrm{d}q_{\textrm{cor,p,in}}^{\textrm{sys},\ell }}{\textrm{d}t}\right) - q_{\textrm{cor,p,in}}^{\textrm{sys},\ell } + q_{\textrm{cor,p}}^{\textrm{sys},\ell }=0\\&R_{\textrm{cor,p}}^{\textrm{sys},\ell }\,q_{\textrm{cor,p}}^{\textrm{sys},\ell }-p_{\textrm{ar}}^{\textrm{sys}}+p_{\textrm{cor,d}}^{\textrm{sys},\ell } + Z_{\textrm{cor,p}}^{\textrm{sys},\ell }\,q_{\textrm{cor,p,in}}^{\textrm{sys},\ell }=0\\&C_{\textrm{cor,d}}^{\textrm{sys},\ell } \frac{\textrm{d}(p_{\textrm{cor,d}}^{\textrm{sys},\ell }-p_{\textrm{v}}^{\ell })}{\textrm{d}t} - q_{\textrm{cor,p}}^{\textrm{sys},\ell } + q_{\textrm{cor,d}}^{\textrm{sys},\ell }=0\\&R_{\textrm{cor,d}}^{\textrm{sys},\ell }\,q_{\textrm{cor,d}}^{\textrm{sys},\ell }-p_{\textrm{cor,d}}^{\textrm{sys},\ell }+p_{\textrm{at}}^{r}=0\\&C_{\textrm{cor,p}}^{\textrm{sys},r} \left( \frac{\textrm{d}p_{\textrm{ar}}^{\textrm{sys},r}}{\textrm{d}t}-Z_{\textrm{cor,p}}^{\textrm{sys},r}\frac{\textrm{d}q_{\textrm{cor,p,in}}^{\textrm{sys},r}}{\textrm{d}t}\right) - q_{\textrm{cor,p,in}}^{\textrm{sys},r} + q_{\textrm{cor,p}}^{\textrm{sys},r}=0\\&R_{\textrm{cor,p}}^{\textrm{sys},r}\,q_{\textrm{cor,p}}^{\textrm{sys},r}-p_{\textrm{ar}}^{\textrm{sys}}+p_{\textrm{cor,d}}^{\textrm{sys},r} + Z_{\textrm{cor,p}}^{\textrm{sys},r}\,q_{\textrm{cor,p,in}}^{\textrm{sys},r}=0\\&C_{\textrm{cor,d}}^{\textrm{sys},r} \frac{\textrm{d}(p_{\textrm{cor,d}}^{\textrm{sys},r}-p_{\textrm{v}}^{\ell })}{\textrm{d}t} -q_{\textrm{cor,p}}^{\textrm{sys},r} + q_{\textrm{cor,d}}^{\textrm{sys},r}=0\\&R_{\textrm{cor,d}}^{\textrm{sys},r}\,q_{\textrm{cor,d}}^{\textrm{sys},r}-p_{\textrm{cor,d}}^{\textrm{sys},r}+p_{\textrm{at}}^{r}=0\\&q_{\textrm{cor,d}}^{\textrm{sys},\ell }+q_{\textrm{cor,d}}^{\textrm{sys},r}-q_{\textrm{cor,d,out}}^{\textrm{sys}}=0 \end{aligned}$$

Using the method described in Sect. 2.3, the following values, except for systemic artery inducatance, are specific to this patient and were fit for the patient’s 0D parameters (Table  2) using patient-specific data. The system artery inductance was chosen based on work done by Hirschvogel et al. (2017).

## Footnotes

1 $\mathcal {F}(\textbf{w},\Gamma _k) = \int _{\Gamma _k} \textbf{w} \cdot \textbf{n} \, dA$ and $\bar{\mathcal {F}}(\cdot ,\Gamma _k) = \mathcal {F} (\cdot ,\Gamma _k) / | \Gamma _k |$ for a vector field $\textbf{w}$ and boundary $\Gamma _k$.
